# Sea turtle fibropapilloma tumors share genomic drivers and therapeutic vulnerabilities with human cancers

**DOI:** 10.1038/s42003-018-0059-x

**Published:** 2018-06-07

**Authors:** David J. Duffy, Christine Schnitzler, Lorraine Karpinski, Rachel Thomas, Jenny Whilde, Catherine Eastman, Calvin Yang, Aleksandar Krstic, Devon Rollinson, Bette Zirkelbach, Kelsey Yetsko, Brooke Burkhalter, Mark Q. Martindale

**Affiliations:** 1 The Whitney Laboratory for Marine Bioscience, Sea Turtle Hospital, University of Florida, St. Augustine, FL 32080 USA; 2 Molecular Ecology and Fisheries Genetics Laboratory, School of Biological Sciences, Bangor University, Bangor, Gwynedd LL57 2UW UK; 3 Department of Biological Sciences, School of Natural Sciences, Faculty of Science and Engineering, University of Limerick, Limerick, Ireland; 4 Department of Biology, University of Florida, Gainesville, FL 32611 USA; 5 The Turtle Hospital, 2396 Overseas Highway, Marathon, FL 33050 USA; 6 Pinecrest Veterinary Hospital, 12125 South Dixie Highway, Pinecrest, FL 33156 USA; 7 Systems Biology Ireland, School of Medicine, University College Dublin, Belfield, Dublin, 4 Ireland

## Abstract

Wildlife populations are under intense anthropogenic pressures, with the geographic range of many species shrinking, dramatic reductions in population numbers and undisturbed habitats, and biodiversity loss. It is postulated that we are in the midst of a sixth (Anthropocene) mass extinction event, the first to be induced by human activity. Further, threatening vulnerable species is the increased rate of emerging diseases, another consequence of anthropogenic activities. Innovative approaches are required to help maintain healthy populations until the chronic underlying causes of these issues can be addressed. Fibropapillomatosis in sea turtles is one such wildlife disease. Here, we applied precision-medicine-based approaches to profile fibropapillomatosis tumors to better understand their biology, identify novel therapeutics, and gain insights into viral and environmental triggers for fibropapillomatosis. We show that fibropapillomatosis tumors share genetic vulnerabilities with human cancer types, revealing that they are amenable to treatment with human anti-cancer therapeutics.

## Abstract (web summary)

David Duffy et al. use a precision-medicine-based approach to study fibropapillomatosis tumors in sea turtles to identify environmental triggers and potential therapeutics. They show that these tumors share genetic similarities with human cancer types, and may be treatable using human anti-cancer therapies.

## Introduction

**Fig. 1:**
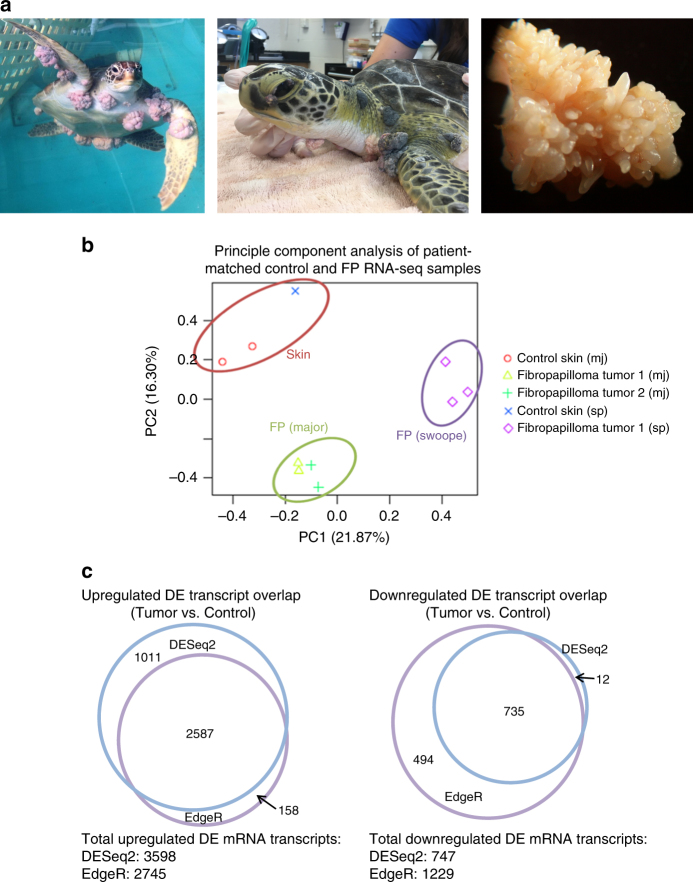
Fibropapillomatosis tumors and differential transcript expression. **a** Fibropapillomatosis-afflicted green sea turtles (*Chelonia mydas*). Left and center: Turtles at the Whitney Sea Turtle Hospital, FL, prior to tumor removal surgery. Right: High magnification image of a fibropapilloma tumor, which was profiled by RNA-seq. **b** Principle component analysis of patient-matched control and fibropapillomatosis RNA-seq samples. Upon admittance to the hospital, Swoope (sp) had a tumor score of class 3 (Hawaii classification system)^105^, and severe (FPI > 185.5) (Southwest Atlantic classification system)^106^, while Major (mj) had a tumor score of class 2^105^, and moderate (FPI = 81.5)^106^. **c** Overlap of transcripts differentially expressed in fibropapillomatosis tumors, independently called by DESeq2 and EdgeR from the RNA-seq data. Area-proportional Venn diagrams were generated using BioVenn (http://www.biovenn.nl/)^107^. Transcripts were considered to be DE if passing the following cut-offs: false discovery rate (EdgeR) or adjusted *p*-value (DESeq2) of <0.05 and log_2_ fold change of >2 or <−2

Wild sea turtle populations, which are already in danger of extinction (http://www.iucnredlist.org/), are under increasing threat from a potentially fatal virulent tumor, fibropapillomatosis (Fig. 1a). These tumors are undermining turtle conservation efforts within Florida (FL, where fibropapillomatosis was first recorded) and circum-globally, with the disease having spread throughout equatorial and subequatorial equatorial regions^1–9^. Green sea turtles (*Chelonia mydas*) are the species most commonly and severely afflicted with fibropapillomatosis (Fig. 1), but it also occurs in all other sea turtle species^4–7,10–12^, and evidence suggests that its geographic range is spreading^6,8,9,13^. Fibropapillomatosis is now being reported from more northern latitudes where it has never been previously recorded^6^. The etiology of fibropapillomatosis is not fully understood, but the disease has a combination of viral and environmental cofactors^5^. A large number of oncogenic viruses have been reported in terrestrial and aquatic wildlife and domestic animals^14,15^. Meanwhile, in humans, the number of cancer incidences identified as attributable to pathogens has tripled in only 4 years^16,17^, now accounting for 15% of all human cancers. Furthermore, emerging infectious disease events in humans are dominated by zoonoses (diseases transmitted to humans from animals, 60.3%), with the majority of these (71.8%) originating in wildlife^18–20^. Many emerging infectious diseases (25–44%) are viral or prion in nature^18,19,21,22^. It is clear that vertebrate virus-borne disease can spread through the marine environment, and that anthropogenic effects (e.g., habitat degradation) are largely responsible for the increased rate in the emergence of infectious diseases^4,18,23–25^. Therefore, a more concerted effort to improve our understanding of pathogen-induced cancers in wildlife, including potential exacerbating effects of human coastal land use, is crucial for enhancing both human and wildlife health.

Despite fibropapillomatosis first being reported in the late 1800s/early 1900s, and having reached panzootic levels in the 1990s^1–3,5,6,26^, practically nothing is known about the host molecular events/genes driving the tumors. Furthermore, the viral dynamics of fibropapillomatosis are poorly understood. Transmissibility of the tumors was experimentally proven in 1995^27^. Fibropapilloma tumors are transmissible to fibropapillomatosis-free turtles via injection of tumor-derived freeze–thawed and filtered sub-cellular fractionations, indicating a viral agent^27^. The inability to culture the responsible virus in a lab setting^28,29^ has meant that Koch’s postulates remain unfulfilled and viral causation has yet to be definitively proven. However, a number of studies have shown fibropapilloma-associated turtle herpesvirus (FPTHV), also known as chelonid herpesvirus 5 (ChHV5) and chelonid fibropapilloma-associated herpesvirus (CFPHV), to be associated with fibropapillomatosis globally^5,10,27,30,31^, and the genome of this virus has been partially sequenced^32,33^. Conversely, high levels of ChHV5 DNA have been detected in clinically healthy marine turtles^30,34^. Recently a step toward completing Koch’s postulates was made with the successful in vitro growth of ChHV5, in tumor cells cultured in a replica complex three-dimensional structure of turtle skin, including both fibropapilloma tumor fibroblasts and turtle keratinocytes^35^.

ChHV5 appears to be a virus upon which oncogenic potential is conferred by a form of external environmental perturbation. This is indicated by the widespread presence of ChHV5 in clinically healthy turtles, including in regions where fibropapillomatosis has never been observed (e.g., Europe) and in non-tumor tissues of fibropapillomatosis-afflicted turtles^7,30,34,36,37^. Furthermore, phylogenetic reconstruction analyses have shown that ChHV5 has been present in sea turtles well before the outbreak of fibropapillomatosis, having co-evolved with its turtle hosts^5,36,38–40^. Current estimates for the virus diverging to becoming specific to marine turtles range from hundreds to millions of years, yet tumors are thought to be only a relatively recent phenomenon^5,36,38–40^. Strong correlative evidence suggests that localized human-induced environmental changes are responsible for conferring oncogenic potential upon ChHV5^5,41–44^. Fibropapillomatosis rates are extremely high in many habitats degraded by human activity, yet almost absent from neighboring more pristine areas^5,43,44^.

Currently, surgical removal is the primary treatment for turtles with fibropapillomatosis, but one study showed that almost 60% of tumors (in 38.5% of cases) regrew post-surgery^7^. The regrowth occurs within an average of 36 days, but since records are only available for the relatively short period of captive rehabilitation, it is likely that the true occurrence of regrowth is much higher. Additionally, there is currently no effective treatment for turtles presenting with internal tumors (which can result in organ failure), detectable by X-ray, magnetic resonance imaging (MRI), or computerized axial tomography (CAT) scanning, with such turtles usually having to be euthanized. Therefore, novel therapies are urgently needed to enhance the rehabilitation outcomes of turtles with fibropapillomatosis.

Although the viral nature of fibropapilloma tumors has been widely, if not conclusively, investigated, the role of the host molecular events in driving these tumors has been largely overlooked. Given that oncogenic viruses play their most critical role in the very early stages of tumor development, primarily in the transformation of host cells, the scope of anti-viral treatments for tackling well-established tumors is severely limited^45^. This fact is highlighted by the hit-and-run hypothesis of viral oncogenic transformation, whereby after inducing the malignant conversion of host cells, viruses are no longer necessary for the maintenance of the malignant state and may no longer be physically present in tumors^46^. Therefore to improve our understanding and treatment of fibropapillomatosis, it is particularly important to examine the molecular mechanisms present in host cells, which drive the growth of established tumors. That turtles tend to present to rehabilitation facilities in the more advanced stages of tumor growth highlights the critical knowledge gap in our understanding of fibropapillomatosis.

Here, we demonstrate the applicability of precision wildlife medicine^4^ to improving animal health and advancing wild animal clinical care. In order to determine the molecular signaling events responsible for driving fibropapilloma tumor growth in green sea turtles (*C. mydas*), we employed precision medicine approaches^47–49^, previously honed in the field of human oncology. This included next-generation Illumina RNA sequencing (RNA-seq) transcriptomic profiling and computationally-based systems-level analysis. We show that in established tumors, there is minimal ChHV5 expression; rather tumors are primarily driven by altered host gene expression, leaving them vulnerable to anti-cancer therapeutics. We thereby advanced the study of host molecular drivers of chelonid fibropapilloma tumors from their current pre-genetic era, bypassing the candidate gene-centric approach straight into the modern genomics era, providing a solid understanding of the basic cell signaling pathways that drive tumor growth, thus enabling the identification of therapeutic targets for fibropapillomatosis treatment.

## Results

### Fibropapillomatosis transcriptomics reveals altered host gene expression

**Fig. 2:**
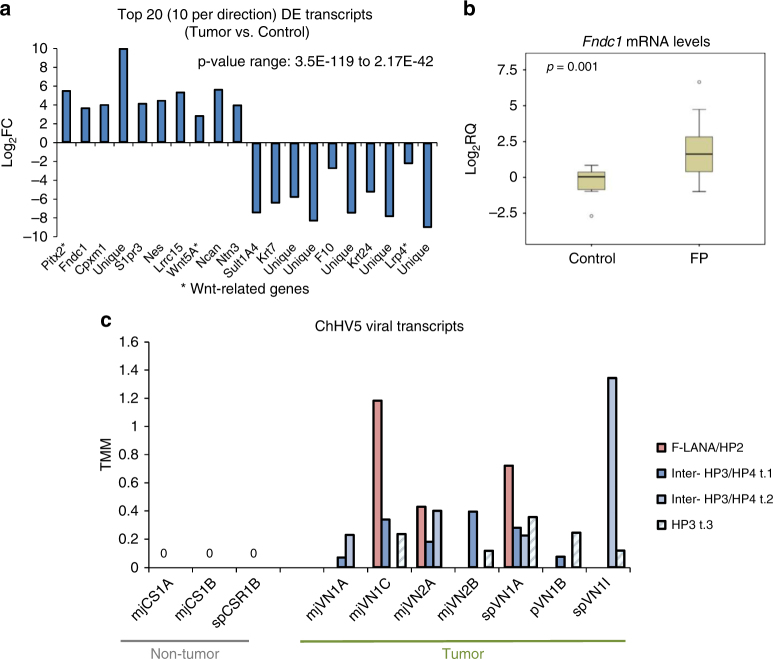
Differentially expressed host (*C. mydas*) and viral (ChHV5) transcripts. **a** Top ten statistically significant upregulated and top ten downregulated transcripts in fibropapillomatosis tumors compared with control tissue. Transcript expression values underlying the differential expression analysis are provided in Supplementary Table 4. Differential expression *p*-values reported are adjusted *p*-values, generated by DESeq2 by a Wald test followed by Benjamini–Hochberg correction. **b** Relative expression of *Fndc1* mRNA in fibropapillomatosis tumor and control samples, as detected by RT-qPCR. Data for 66 tumor samples and 14 control samples. The *p*-value was calculated by *t*-test. **c** Expression levels (trimmed mean of *M* values, TMM) of ChHV5 viral RNA de novo transcripts across the ten RNA-seq samples. Related transcript variants are denoted by t.1, t.2, or t.3

We transcriptomically profiled tumor tissue, surgically removed by laser resection as part of the turtle’s rehabilitative care, and compared global gene expression to that of biopsies from non-tumor areas of the same turtles. Ten RNA samples from two juvenile green turtles (*C. mydas*), which had stranded in northern FL were used for sequencing (Fig. 1b). The early development phase of fibropapilloma tumors is associated with proliferation of epidermal cells, while later growth is associated with proliferation of the dermal layer^5^. Therefore, in order to obtain representative untransformed control tissue, we profiled skin punch biopsies containing epidermis, dermis, and subcutaneous tissue. Principle component analysis of the RNA-seq data confirmed that non-tumor samples grouped separately from tumor samples (Fig. 1b). A de novo transcriptome was assembled from the sequencing reads using Trinity v2.1.1^50^. The transcriptome was generated using Trinity’s unguided function, to ensure that any tumor-specific or viral transcripts present would not be filtered out during transcriptome generation, and given that currently only a draft *C. mydas* genome exists. The de novo transcriptome consisted of 266,069 transcripts, with an N50 length (the largest length such that 50% of all base pairs are contained in contigs of this length or larger) of 1562 bp. Pair-wise comparison of the tumored and non-tumored tissue revealed that 4345 transcripts were differentially expressed (DESeq2, adjusted *p*-value cut off of 0.05 and with a log2 ± 2 fold change applied) (Fig. 1c), with the majority of these (83%) being upregulated in the tumors (Supplementary Figure 1A, B). When EdgeR was used to independently call differentially expressed (DE) transcripts, there was a strong overlap between the transcripts called as DE between DESeq2 and EdgeR (Fig. 1c), adding confidence that these are genuine DE transcripts and not artifacts of the computational analysis. We confirmed the upregulation of *Fibronectin Type III Domain Containing 1* (*FNDC1*, a cancer-associated gene^51^), a top DE transcript (Fig. 2a), in an extended panel of fibropapillomatosis tumor samples (14 control and 67 tumor samples) by qPCR (Fig. 2b).

### RNA-seq reveals a paucity of ChHV5 viral transcripts within tumors

Having confirmed the differential expression of host genes within the tumors, we next examined ChHV5 viral expression by blasting the de novo transcriptome against the ChHV5 partial genome^33^ [GenBank accession number: HQ878327.2]. Although ChHV5 has been associated with fibropapillomatosis and ChHV5 DNA is readily detectable within tumors^5,31^, there was an almost complete lack of viral transcripts within the RNA-seq samples, with only four ChHV5 transcripts being detected, three of which were related transcript variants (Fig. 2c, Supplementary Table 1). Interestingly, no viral transcripts were detected in the non-tumor tissue samples, being specific to the tumor samples (Fig. 2c). However, even in the tumors, there was a paucity of viral transcripts expressed, with the transcripts related to only two regions of the genome; the *F-LANA/HP2* and the *HP3/HP4* regions (Fig. 2c). Expression levels of these transcripts were also relatively low (viral expression range: 0–1.344 trimmed mean of *M* values (TMM). The range of expression for all transcripts [viral and host] was 0–125,636.734 TMM, with the average expression level of all transcripts across samples being 4.608 TMM.) Within the ChHV5 genome, the coding region of the *F-LANA* and *HP2* genes overlap, one transcript from the RNA-seq mapped to this region (Fig. 2c and Supplementary Table 1). However, for the *HP3/HP4* region, three related but distinct transcript variants were called (Supplementary Table 1), one within the *HP3* gene and two mapping to the region between the *HP3* and *HP4* genes (Fig. 2c and Supplementary Table 1). Due to a genomic duplication event^33^, all four viral transcripts detected map to two locations within the ChHV5 genome, at both the terminal and internal repeat regions (Supplementary Table 1).

To confirm the paucity of viral transcripts independently of our de novo transcriptome, we also aligned the reads for each sample against the ChHV5 genome. As with the de novo transcriptome analysis, direct mapping to the ChHV5 genome also showed viral reads predominantly aligning to the terminal and internal repeat region around the *F-LANA*, *HP2*, *HP3*, and *HP4* genes (Supplementary Figure 1C). *F-LANA* is a latency-associated nuclear antigen gene, which promotes viral latency by suppressing viral transcription and facilitating DNA replication^52^. The detection of the *F-LANA* expression along with the paucity of other ChHV5 transcripts suggests that ChHV5 is latent within these tumors. Viral latency would indicate that any causative role for ChHV5 in fibropapillomatosis tumorigenesis occurs early, and that the virus does not continue to drive the growth of established tumors.

### Fibropapillomatosis has a neuronal signature and similarity to human cancer

**Fig. 3:**
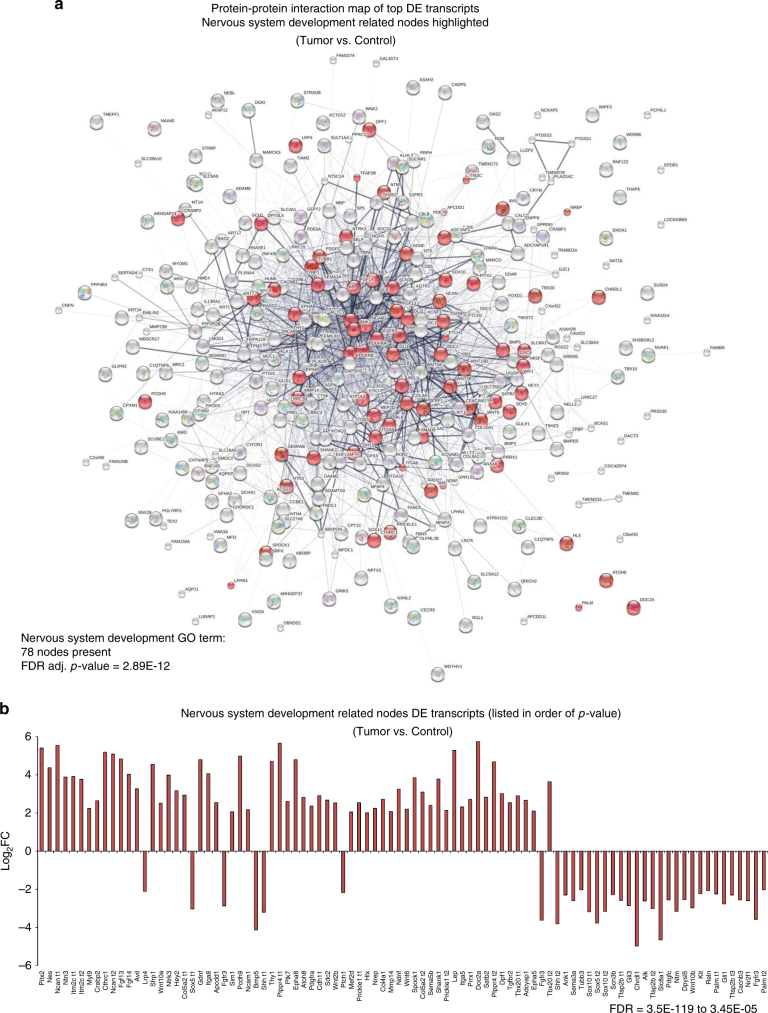
Transcripts differentially expressed in fibropapillomatosis are associated with nervous system development. **a** Protein–protein interaction map of the top differentially expressed transcripts (RNA-seq) with homology to characterized human genes. Generated from the top 600 (300 per direction of regulation) differentially expressed transcripts. Nervous system development-related nodes are highlighted by red shading. Network generated by the STRING database^54^ (v10.5, http://www.stringdb.org), with the inbuilt KEGG pathway enrichment analysis tool applied to this network. **b** Fold change in expression of the differentially expressed transcripts (RNA-seq) related to nervous system development (KEGG), listed in order of false discovery rate (FDR, KEGG pathway enrichment analysis tool). Transcript expression values underlying the differential expression analysis are provided in Supplementary Table 4

To determine the molecular mechanisms driving fibropapillomatosis tumors, we took the top 600 (300 per direction of regulation, Supplementary Table 2) statistically significant DE transcripts (DESeq2 analysis, adjusted *p*-value cut-off of 0.05 and with a log2  ±  2 fold change applied, the adjusted *p*-value ranges of the 300 upregulated and downregulated transcripts were 3.52E−119 to 3.15E−14 and 1.33E−105 to 3.35E−05, respectively), determined their closest characterized homolog using BLASTn (https://blast.ncbi.nlm.nih.gov/Blast.cgi)^53^, and performed systems level analysis. Network analysis of these transcripts (STRING database v10.5, http://www.stringdb.org/)^54^ revealed a highly interconnected network, which was strongly enriched for neuronal-related genes (Fig. 3a, b), with nervous system development and neurogenesis gene ontology (GO) terms being strongly enriched (FDR *p*_adj._ = 2.89E−12 and 2.03E−11, respectively).

**Fig. 4:**
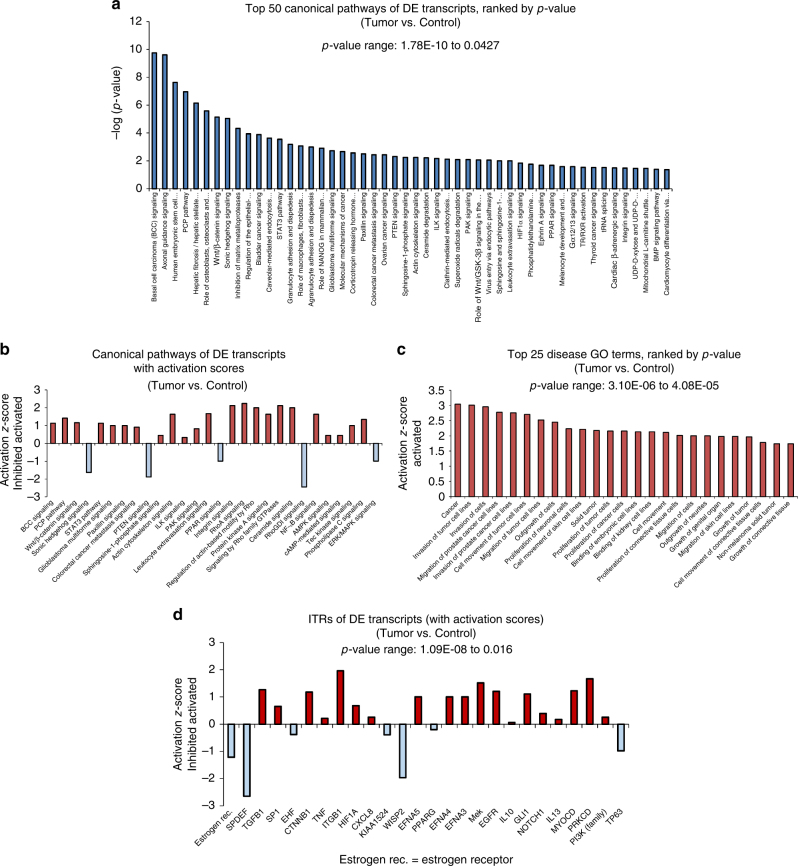
Pathway, disease gene ontology (GO) term, and transcriptional regulator analysis of the top 600 transcripts differentially expressed in fibropapillomatosis tumors. **a** Top 50 canonical pathways of the fibropapillomatosis tumor differentially expressed transcripts (RNA-seq), as detected by IPA, ranked by *p*-value (calculated by right-tailed Fisher’s Exact Test, with Benjamini–Hochberg correction). **b** Activation/inhibition *z*-scores of the canonical pathways of the fibropapillomatosis tumor differentially expressed transcripts, as detected by IPA. **c** Activation *z*-scores of the top 25 disease-associated GO terms of the fibropapillomatosis tumor differentially expressed transcripts (RNA-seq), as detected by IPA, ranked by *p*-value (calculated by right-tailed Fisher’s Exact Test, with Benjamini–Hochberg correction). **d** Activation/inhibition *z*-scores of the inferred transcriptional regulators (ITRs) of the fibropapillomatosis tumor differentially expressed transcripts (RNA-seq), as detected by IPA, ranked by *p*-value (calculated by right-tailed Fisher’s Exact Test, with Benjamini–Hochberg correction). Legend of *x*-axis labels for Fig. 3a–c is also provided in Supplementary Table 5

We next performed Ingenuity Pathway Analysis (IPA, https://www.qiagenbioinformatics.com/products/ingenuity-pathway-analysis/)^55^, which revealed that fibropapilloma tumors share a striking similarity to a number of human cancers (Fig. 4a–c). Human basal cell carcinoma (BCC) signaling was the top-ranked signaling pathway in fibropapillomatosis tumors when compared with non-tumored control tissue (*p* = 1.77E−10, Fig. 4a), with BCC signaling being activated (Fig. 4b). Mirroring the network analysis findings, axonal guidance signaling was the second most significant pathway identified (*p*-value = 2.45E−10) (Fig. 4a). In addition to their role in neuronal development and neuronally-derived tumors (e.g., medulloblastoma and neuroblastoma), axonal guidance genes have been shown to be involved in driving tumorigenesis in a range of other non-neuronally-derived human cancer types such as pancreatic cancer^56^. Human embryonic stem cell pluripotency signaling was the third most highly ranked pathway (*p* = 2.34E−08), in line with the fibropapillomatosis tumors consisting of less differentiated more stem-like cells, as is the case in human tumors^57,58^.

Cancer and neuronal disease terms also topped the disease GO terms analysis (Fig. 4c and Supplementary Figure 1C), with cancer growth, invasion, and metastasis terms being activated, while cell death and apoptosis terms were inhibited (Supplementary Figure 1C), again consistent with fibropapillomatosis tumors tending to have rapid growth rates, and a propensity for tens to hundreds of tumors to develop on a single infected individual. Strikingly, of the 334 unique genes (from the top 600 DE transcripts) with homology to human genes, almost all of them (321 genes) are associated with human cancer (Fig. 4c, *p*-value = 3.10E−06). This includes the upregulation of genes such as *ITGA5* (Supplementary Table 2), which are involved in virally-mediated angiogenesis promotion in Kaposi’s sarcoma^59^.

IPA inferred transcriptional regulator (ITR) analysis, which matches observed DE genes to known patterns of gene regulation by specific transcriptional regulators^55^, revealed a highly interconnected network of regulators, governing the gene expression changes seen in fibropapillomatosis (Supplementary Figure 1D). The ITR analysis also suggested a number of potential novel therapeutics for fibropapillomatosis treatment such as β-estradiol, or inhibitors of a number of pathways including TGFβ, Wnt, MAPK, and EGFR (Fig. 4d).

### Wnt and SHH signaling regulate fibropapillomatosis,  which correlates with UV exposure

**Fig. 5:**
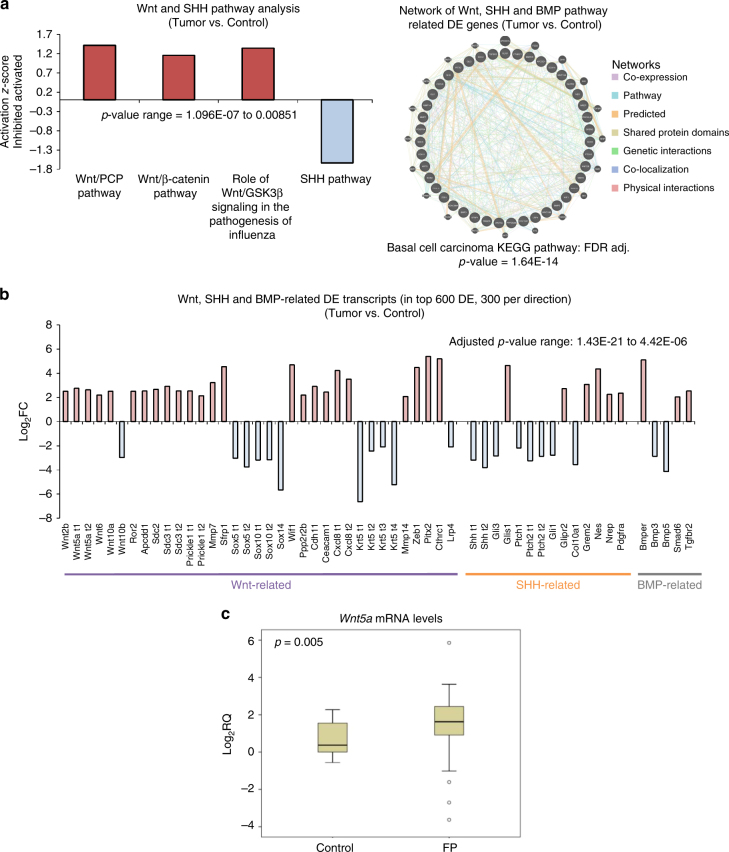
Wnt, SHH, and BMP pathways in fibropapillomatosis tumors. **a** Left: Activation/inhibition *z*-scores of Wnt- and SHH-related IPA pathway analysis findings of the fibropapillomatosis tumor differentially expressed transcripts (RNA-seq), *p*-values calculated by right-tailed Fisher’s Exact Test, with Benjamini–Hochberg correction. Right: Interaction map of the 43 Wnt, SHH, and BMP pathway component and target genes differentially expressed in fibropapillomatosis tumors (RNA-seq). Image generated using GeneMANIA (v3.5.0, http://genemania.org/)^104^. In addition to the 43 DE genes (nodes with stripped shading), 20 closely functionally-related network components are also shown (nodes with uniform shading). Basal Cell Carcinoma KEGG pathway score of the DE components of the network is shown below the network. **b** Fold change in expression of the differentially expressed transcripts (RNA-seq) of Wnt, SHH, and BMP pathway-related genes. When more than one transcript of a gene was present in the top 600 DE genes (300 per direction), each transcript is denoted by t1, t2, t3, etc. Differential expression *p*-values reported are adjusted *p*-values, generated by DESeq2, by a Wald test followed by Benjamini–Hochberg correction. Transcript expression values underlying the differential expression analysis are provided in Supplementary Table 4. **c** Relative expression of Wnt5a mRNA in a panel fibropapillomatosis tumor and control samples, as detected by RT-qPCR. Data for 66 tumor samples and 13 control samples. The *p*-value was calculated by *t*-test

Our systems-level analysis identified shared molecular networks between sea turtle fibropapillomatosis and human BCC, including Wnt, BMP, and SHH signaling. While being involved in a variety of other cancer types^60–66^, Wnt and sonic hedgehog (SHH) signaling are a part of a shared regulatory network in BCC and are differentially activated in fibropapilloma tumors (Fig. 5a, b). We confirmed that *Wnt5a* was differentially expressed in a large panel of fibropapilloma tumors, primarily being upregulated (Fig. 5c). *Wnt5a* is an important regulator of both the Wnt/PCP and Wnt/β-catenin pathways, playing a prominent role in neuronal stem cells and nervous system development^67,68^, and is an important regulator of tumorigenicity of many human cancers, including neuronal derived ones^62,67–72^.

**Fig. 6:**
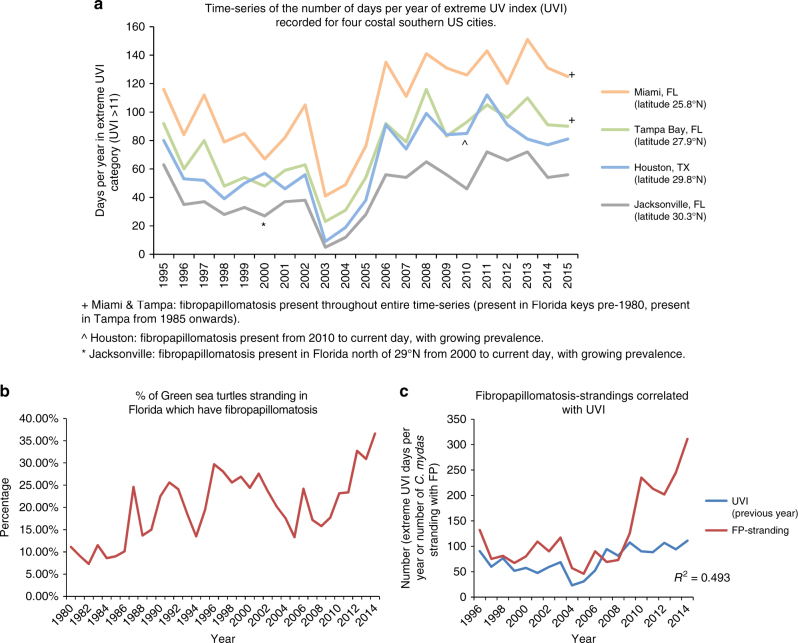
Putative fibropapillomatosis prevalence and ultraviolet (UV) radiation exposure link. **a** Number of days annually in the extreme UV index (UVI) category recorded in Miami, Tampa Bay, Houston, and Jacksonville (data obtained from: http://www.ftp.cpc.ncep.noaa.gov/long/uv/cities/). Dates for the occurrence of fibropapillomatosis in the vicinity of each UVI sampling site obtained from Hargrove et al.^6^. **b** Annual percentage of green sea turtles stranding in FL afflicted with fibropapillomatosis, obtained from Foley et al.^73^. **c** Yearly averages of Jacksonville, Miami, and Tampa Bay UVI extreme days annually, vs. annual fibropapillomatosis-afflicted green turtle strandings in FL. Correlation between fibropapillomatosis strandings and UVI conducted using *R*^2^ linear regression analysis

As BCC is a skin cancer with both genetics and ultraviolet radiation (UV) exposure as risk factors, we investigated whether any correlation between fibropapillomatosis incidence and high UV exposure levels exists. Historical daily UV index (UVI) data, dating from 1995 to 2015, were available for three coastal cities in FL: Jacksonville (north FL), Tampa Bay (central FL), and Miami (south FL) (http://www.ftp.cpc.ncep.noaa.gov/long/uv/cities/ and http://www.cpc.ncep.noaa.gov/products/stratosphere/uv_index/uv_annual.shtml). Despite fibropapillomatosis’s rapid circum-global spread (including the Caribbean, Hawaii, Australasia, India, and Africa), fibropapillomatosis has been much slower to spread to more temperate latitudes, with northern FL (north of latitude 29°N) remaining fibropapillomatosis-free until 2000, despite fibropapillomatosis being present in the FL Keys since before 1913^1–3,6^. Therefore, while fibropapillomatosis was present in the Miami and Tampa areas for the entire duration for which UVI recordings are available, the Jacksonville data cover the period of the spread of fibropapillomatosis into northern FL (Fig. 6a). There was a clear trend from north to south in the number of days annually with extreme UV exposure levels (UVI > 11), and the number of extreme UVI days has increased from 1995 to 2015 across all three FL sites. Between 1995 and 2015, both the total number of green sea turtles (*C. mydas*) stranding in FL exhibiting fibropapillomatosis and the percentage of all stranded *C. mydas* in FL, which are afflicted with fibropapillomatosis has grown^73^ (Fig. 6b,c). The increasing incidence of fibropapillomatosis in FL is in line with global fibropapillomatosis incidence trends^6^. We next performed a linear regression analysis to determine whether there was any correlation between the number of *C. mydas* stranding with fibropapillomatosis each year and the number of days of extreme UVI (average of all three sites) in the previous year (Fig. 6d). UVI levels from the previous year were used due to the recognized lag between UV exposure and skin cancer development^74^. The analysis revealed that there was a mild positive correlation between UVI exposure and the numbers of stranded fibropapillomatosis-afflicted turtles (*R*^2^ = 0.493), suggesting that UV exposure may also be a risk factor for fibropapillomatosis and a potential limiting factor accounting for the relatively slow northward spread of fibropapillomatosis. The observed level of correlation is relatively high, considering that fibropapillomatosis is a multifactorial disease with viral and other environmental factors likely contributing to disease progression^5^.

### 5-FU post-surgical treatment mitigates fibropapillomatosis tumor regrowth

We revealed that fibropapillomatosis shares molecular characteristics with human BCC, for which a number of effective therapies exist including the topical application of fluorouracil (5-FU) onto affected skin regions. While 5-FU is used in a number of human cancers, topical application is a first line treatment in BCC^75^. Sixty percent of fibropapillomatosis tumors regrow after surgical removal^7^, with surgery being the only widely employed treatment option. As is the case in human oncology, adjunct drug/chemotherapy treatment post-surgery is likely to dramatically reduce tumor regrowth rates, thus the utility of 5-FU as an adjunct therapy in fibropapillomatosis was assessed in eye tumors.

**Fig. 7:**
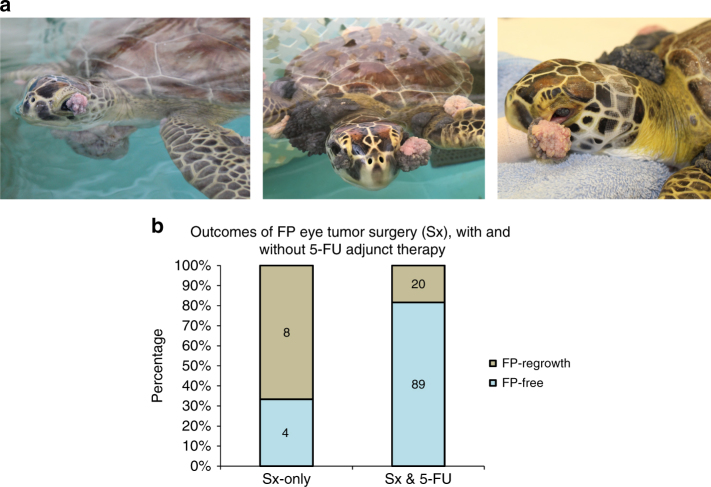
Fibropapillomatosis eye tumor regrowth rates in the presence and absence of adjunct 5FU treatment. **a** Green sea turtles afflicted with fibropapillomatosis eye tumors. **b** Fibropapilloma eye tumor regrowth rates, when treatment consists of surgical removal only, or surgical removal followed by 8 weeks of topical 5-FU treatment. Total *n* = 121 turtles, with the number of turtles per condition inserted within the relevant section of each bar

Fibropapillomatosis tumors commonly occur in the eye region, often arising from the conjunctivae or cornea^5,7^ (Fig. 7a). Eye tumors can grow to a considerable size, obscuring vision. Given the smaller surface area of eye tumors and the high level of debilitation caused by them, these tumors are particularly suited to topically delivered post-surgery chemotherapy to help prevent tumor regrowth. Sixty-seven percent of turtles whose eye tumors (ocular and periocular) were treated with surgery-only experienced eye tumor regrowth (Fig. 7b, Supplementary Table 3), this level of regrowth is in line with the rates seen across all fibropapillomatosis tumors (regardless of body location) in other studies, i.e., 60% of tumors re-growing^7^. However, when turtles received 5-FU post-surgery (8 week 5-FU course, treated twice daily with 1% topical 5-FU drops), only 18% of turtles experienced eye tumor recurrence (Fig. 7b). The duration of post-treatment follow-up varied as dictated by the individual circumstance of each turtle (Supplementary Table 3), but was between 2 and 12 months.

## Discussion

The increased rate of emerging diseases, and their exacerbating negative effects on already stressed wild populations, poses an additional burden on already elevated rates of population reduction and species extinction^4,18,23–25,76–81^. The application of omic technologies and human precision medicine approaches can rapidly help alleviate the burden of such diseases and assist in identifying causative factors, ultimately improving species conservation^1^. To this end, we applied next-generation transcriptomic profiling and systems level analysis to fibropapillomatosis tumors, revealing evolutionarily conserved molecular mechanisms between fibropapillomatosis and human cancer types such as BCC and neuronally-derived tumors. BCC itself is known to have neuronal links, including the SHH pathway and correlation between different BCC sub-types and neuronal differentiation markers^82^. The neuronal signature of fibropapillomatosis tumors suggests either extensive innervation in these tumors, a previously unappreciated role of neuronal blast cells in the origin of fibropapillomatosis tumors, or at least the involvement of neuronal-related genes in fibropapillomatosis growth. ChHV5 has previously been shown to reside in sea turtle neuronal cells^37^, supporting a neuronal origin hypothesis. To mitigate against any potential cell type-dependent gene expression biases and confirm our findings, our fibropapillomatosis sequencing data should be compared against additional *C. mydas* tissue-specific transcriptomic profiles as they become available.

Interestingly, we detected a paucity of ChHV5 transcripts in our sequencing data. While, this is in line with suggestions that ChHV5 remains latent in established tumors^5,37^ and is therefore largely transcriptionally inactive, analysis of a wider variety of fibropapillomatosis tumor transcriptomes is required to confirm this finding. Our tumor profiling also suggests a number of potentially promising novel therapeutic approaches. Adjunct 5-FU treatment dramatically reduced the post-surgery regrowth rate of eye tumors, demonstrating potential to reduce rehabilitation costs and burdens on individual turtles by negating the need for additional rounds of surgery, and shortening the rehabilitation period. The ability of 5-FU to successfully reduce fibropapillomatosis tumor recurrence rates reinforces the power of genomics-era analysis to rapidly identify novel therapeutic options for fibropapillomatosis treatment. While, 5-FU proved effective for adjunct fibropapillomatosis eye tumor therapy, it warrants further investigation in an independent, controlled clinical study. Such a study should also investigate 5-FU as a treatment for tumors in other body locations. However, as 5-FU is a cytotoxic drug, and in BCC, it is only effective against low-grade tumors^75^, more target therapies should also be trialed. Our analysis also predicts a number of pathways for which more targeted therapies exist, which are likely to have applicability as both adjunct and first-line treatments. SHH and Wnt signaling form an interconnected regulatory node in fibropapillomatosis tumors, the targeting of which could potentially prove effective for fibropapillomatosis treatment. Vismodegib (trade name, erivedge), an inhibitor of the SHH receptor *Smoothened* (*SMO*), is already in use clinically for the treatment of BCC^83^, while a number of Wnt modulating therapies are being actively pursued as anti-cancer treatments, currently at the clinical trial stage^84^. Other targets include MAPK, TGFβ, and estrogen signaling. These are already used in the treatment of a number of human cancers, including neuronally-derived tumors, and their effectiveness as fibropapillomatosis therapeutics should be further investigated^85–90^. To fully achieve the promise of precision medicine and move toward more targeted treatment, newer generation therapeutics highlighted by our findings, such as vismodegib, should be trialed as anti- fibropapillomatosis treatments.

The shared molecular drivers between fibropapillomatosis and BCC suggested a putative role of UV radiation as one of the environmental co-triggers of fibropapillomatosis. Interestingly, Wnt and Metalloprotease signaling are two of the top DE pathways in fibropapillomatosis tumors. These pathways have been shown to be regulated by UV exposure in human skin cells and canine cornea, and mediate UV-induced abnormal morphogenesis in Hydra^91–94^. UV exposure has also been linked to systemic immunosuppression^95^. Immunosuppression also occurs in turtles with fibropapillomatosis, although it has not yet been proven if this is a cause or a consequence of fibropapillomatosis^5^. Taken together, further investigation into interactions between UV exposure, regulation of host gene expression including immune-related genes, and ChHV5 viral load are highly warranted. The correlation between fibropapillomatosis incidence and extreme UV conditions suggests that UV may be another risk factor for fibropapillomatosis. Fibropapillomatosis is most prevalent during green sea turtles’ juvenile inshore life-stage when they inhabit more UV-exposed waters^5,10^. Even in an artificial transmission setting, fibropapillomatosis tumors arose simultaneously, regardless of the time of initial inoculation, only after temperatures increased^27^. The authors attributed this to potential effects of water temperature^27^, though this seasonality is also a proxy for UV exposure. While our analysis focused on FL, UV exposure is also altering globally as a result of climate change^96^. In the case of fibropapillomatosis, UV exposure could be directly involved in oncogenic transformation, as is the case for human skin cancers, or alternatively could be acting through indirect mechanisms. For instance, increased UV could contribute to immunosuppression resulting in reduced control of ChHV5 infections^95^, increased frequency of algal bloom events, which have been correlated with fibropapillomatosis occurrence^5,42–44,97^, or UV radiation, which can alter the chemical composition of contaminants in inshore waters into carcinogenic forms^98,99^. Future research is required to determine whether UV exposure is causally linked to fibropapillomatosis tumor development, and if so, via which mechanism. The genomic profiling reported here has improved our molecular understanding of fibropapillomatosis by revealing the host signaling pathways involved in tumors and indicating novel rehabilitation strategies for fibropapillomatosis-afflicted sea turtles. Additionally, this study demonstrates the power of precision medicine approaches^47,48^ to tackle rare or understudied diseases for which there is limited background genetic knowledge, and provides a proof-of-principle study for the emerging field of precision wildlife medicine^4^.

## Methods

### Tissue sampling

Sampling was carried out under permit number MTP-17–236 from the Florida Fish and Wildlife Conservation Commission and with ethical approval from the University of Florida Institutional Animal Care and Use Committee (IACUC). Fibropapilloma tumors were surgically removed by laser resection as part of the turtle’s rehabilitative care. All samples were obtained from juvenile *C. mydas*, as this is the stage most afflicted by fibropapillomatosis^5,10^. As juveniles, the sex of an individual is not readily determinable. However, one of the turtles whose tumors were sequenced, Swoope (sp, patient ID: 02-2015-Cm), was later determined to be male during necropsy, after being euthanized due to the presence of internal tumors. Global gene expression of the tumors was compared with that of non-tumored areas of the same turtles, obtained by 4 mm punch biopsies during the tumor removal surgery. Non-tumored sites were selected by gross examination of the region by the attending veterinarian and confirmed visually to be tumor-free normal skin regions and not bordering any tumorous regions by the attending vet technicians and researchers. Samples were stored in RNA-later (Qiagen) at −80 °C, according to manufacturer’s instructions, for up to 3 months.

### RNA extraction and RNA-seq

Total RNA was extracted using an RNeasy Kit (Qiagen) according to manufacturer’s instructions. DNA was digested with DNA-free Kit (Applied Biosystems). RNA quality was checked by RT-qPCR (as below) and on a 2100 Bioanalyser (Agilent) using a Eukaryote Total RNA Nano Chip, samples’ RIN value range 8.1–9.8. Ten RNA samples, comprising seven fibropapillomatosis tumor samples (shoulder and neck cutaneous verrucous fibropapilloma tumors) and three non-tumor samples, from two juvenile green turtles (*C. mydas*), which had stranded in Northern FL were used for sequencing (Fig. 1b). Sequencing libraries were generated from 500 ng of total RNA using the NEBNext Ultra RNA Library Prep Kit for Illumina (New England Biolabs), including ployA selection, according to manufacturer’s protocol. Size and purity of the libraries were analyzed on a Bioanalyser High Sensitivity DNA chip (Agilent). Libraries were paired-end sequenced with a read length of 75 bp a HiSeq 3000 (Illumina). ERCC Spike-In Mix (ThermoFisher) was used as an internal control, 2 µL of 1:400 diluted ERCC Spike-In Mix with 500 ng of total RNA input.

Reads were trimmed with Trim Galore (https://www.bioinformatics.babraham.ac.uk/projects/trim_galore/) to remove low-quality ends from reads in addition to adapter removal. A de novo transcriptome was constructed from the sequencing data using Trinity v2.1.1^50^, specifying a minimum contig length of 300. Transcript abundance was quantified with RSEM using bowtie2 for the alignment using the align_and_estimate_abundance.pl script available through the Trinity toolkit. A matrix of gene counts (genes.counts.matrix) and a matrix of TMM-normalized expression values (TMM.EXPR.matrix) was constructed in RSEM using the abundance_estimates_to_matrix.pl script available through the Trinity toolkit^100,101^. A PCA plot (Fig. 1b) was generated using the PtR script in the Trinity toolkit.

Prior to differential expression analysis, the genes.counts.matrix was processed with the RUVseq Bioconductor package^102^ using the RUVs method to remove low abundance genes, normalize the RNA-seq data, and remove unwanted variation. The RUVseq-processed matrix was then used to identify DE transcripts using both the EdgeR and DESeq2 Bioconductor packages. The resulting lists of DE genes were sorted and filtered to include only those transcripts with false discovery rate (EdgeR) or adjusted *p*-value (DESeq2) of <0.05 and log_2_ fold change of >2 or <−2. A list of upregulated and downregulated transcripts that overlapped from both analysis methods was generated and used to create area-proportional Venn diagrams of overlap using BioVenn (http://www.biovenn.nl/) (Fig. 1c).

The retrieved gene lists were analyzed for overrepresented pathways, biological functions, and upstream regulators using IPA (Ingenuity Systems, www.ingenuity.com). *p*-Values reported for IPA results are generated by IPA using a right-sided Fisher exact test for over-representation analysis, Benjamini–Hochberg correction for multiple hypothesis testing correction, and a *z*-score algorithm for upstream regulator analysis, *p*-values < 0.05 were considered significant. For the systems-level analysis, the *C. mydas* DE transcripts were annotated to their closest characterized human homolog. Human annotation was used to enable the most comprehensive systems-level analysis, as human genes have been the most extensively annotated and characterized.

To detect ChHV5 transcripts independently of our de novo transcriptome analysis, trimmed reads (see above) were aligned to the ChHV5 partial genome [GenBank accession number: HQ878327.2] using HISAT2 (https://ccb.jhu.edu/software/hisat2/index.shtml)^103^. Aligned non-repetitive reads were blasted (megablast, https://blast.ncbi.nlm.nih.gov/Blast.cgi) against the NCBI nucleotide collection. These reads were specific to ChHV5 and their location within the ChHV5 genome were visualized using Blast’s alignment graphics function (https://goo.gl/YVQrkc).

*Additional software tools and datasets*: IPA software was used for the ITR, pathway, and GO analysis. String (www.string-db.org) was used to generate protein–protein interaction networks, and the KEGG pathway enrichment analysis tool in String was also applied to these networks. Area-proportional Venn diagrams were generated using BioVenn (http://www.biovenn.nl/). GeneMANIA (v3.5.0, http://genemania.org/)^104^ was used to generate multiple association networks. UVI data was obtained from publicly available NOOA datasets (http://www.ftp.cpc.ncep.noaa.gov/long/uv/cities/ and http://www.cpc.ncep.noaa.gov/products/stratosphere/uv_index/uv_annual.shtml). Stranding data was obtained from Foley et al.^73^, with the raw data points generously provided directly to us by Dr. Allen Foley.

### RT-qPCR

RNA was extracted as above. Sixty-six tumor and 14 non-tumor RNA samples from five juvenile green turtles (*C. mydas*), which had stranded in Northern FL were used for cDNA was synthesis using a QuantiTect Reverse Transcription Kit (Qiagen) including genomic DNA digestion. A LightCycler480 Instrument II (Roche) and LightCycler480 SYBR 1 Master reagents (Roche) were used according to the manufacturer’s protocol. Cycling parameters were as follows: 95 °C 10 min, 45 cycles of 95 °C 10 s, 60 °C 20 s, 72 °C 20 s and a melting curve. Primer sequences are provided in Table 1. Gene expression was normalized to the expression of *β-actin*^31^ with *Rplo* (Table 1) as a second endogenous control. Technical replicates for every sample were also performed. Upon admittance to the hospital, the turtles used in the qPCR study had a tumor score range of 2–3 (Hawaii classification system)^105^, and a Southwest Atlantic fibropapillomatosis (FPS_SWA_) tumor score range of mild to severe (fibropapillomatosis index, FPI, range, 0.6 to >185.5) (Southwest Atlantic classification system)^106^.

**Table 1:** *Chelonia mydas* primer sequences for RT-qPCR

Primers list	Primer sequence	Product size
*β-actin* Fwd^31^	TGGTACAGTCTCCCATTCCA	232 bp
*β-actin* Rev^31^	AGGCATACAGGGACAACACA	
*Rplo* Fwd	CTGTGGCTGTGGAGACTGAA	187 bp
*Rplo* Rev	CCAAATCCCATGTCCTCATC	
*Fndc1* Fwd	ATTACTGCCCGCTTCCCAAA	81 bp
*Fndc1* Rev	GCAAAAGCTGGTGTGTCTGG	
*Wnt5a* Fwd	ACCAAACAGTGTGCTCTGGT	60 bp
*Wnt5a* Rev	CAGTGAAACAGCTGCAGTGG	

### Data availability

The RNA-seq data was deposited at NCBI (https://www.ncbi.nlm.nih.gov/) under BioProject ID: PRJNA449022 (http://www.ncbi.nlm.nih.gov/bioproject/449022), while the Transcriptome Shotgun Assembly project has been deposited at DDBJ/EMBL-EBI/GenBankunder the accession GGMX00000000. The version described in this paperis the first version, GGMX01000000.

## Electronic supplementary material

Supplementary Information
