# Supplementary material for: Sea turtle fibropapilloma tumors share genomic drivers and therapeutic vulnerabilities with human cancers
Source: Commun Biol. 2018 Jun 7;1:63. doi: 10.1038/s42003-018-0059-x (PMC6123702; doi:10.1038/s42003-018-0059-x)
Supplement: Supplementary file 1 — Supplementary Information [file 42003_2018_59_MOESM1_ESM.pdf]

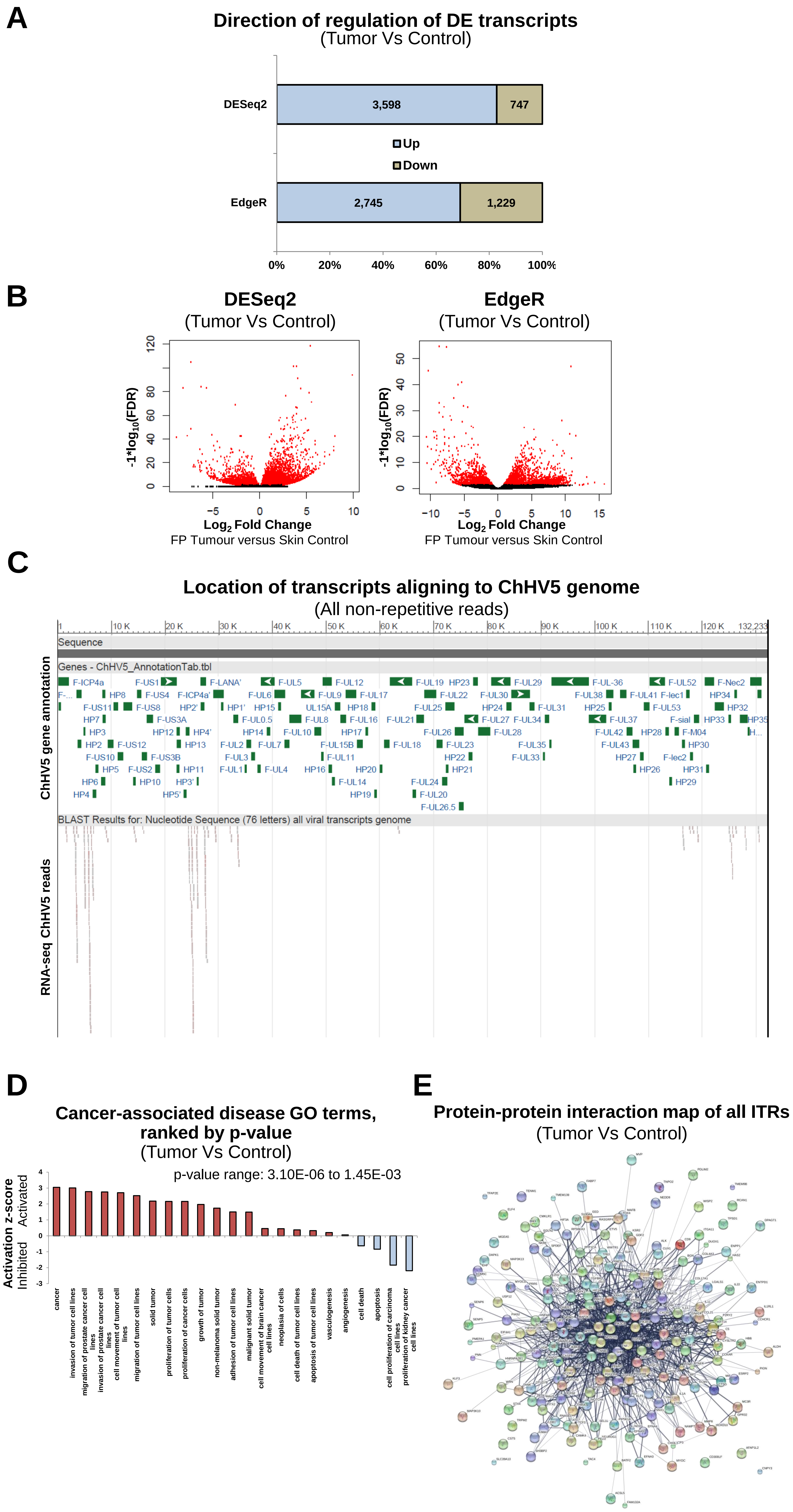

Supplementary Figure 1. Additional sea turtle fibropapilloma RNA-seq analysis. A) The proportion of upregulated and downregulated transcripts (RNA-seq) between fibropapillomatosis tumor and control skin samples, detected by DESeq2 and EdgeR, adj. p-value/FDR cut-off 0.05 and log_2FC of >2 or <-2. **B)** Volcano plots of differentially expressed transcripts (RNA-seq) between fibropapillomatosis tumor and control non-tumor samples, detected by DESeq2 and EdgeR, adj. p-value/FDR cut-off 0.05. **C)** Plot of ChHV5 genomic locations of all non-repetitive reads from the ten RNA-seq samples which aligned to the ChHV5 genome as determined by the HISAT2 aligner. Plot generated using NCBI blast's visualization tool (https://blast.ncbi.nlm.nih.gov/Blast.cgi?PROGRAM=blastn&PAGE_TYPE=BlastSearch&LINK_LOC=blasthome). **D)** Activation/inhibition z-scores of cancer-associated disease GO terms of the fibropapillomatosis tumor differentially expressed transcripts (RNA-seq), as detected by IPA, ranked by p-value (calculated by right-tailed Fisher's Exact Test, with Benjamini-Hochberg correction). Legend of x-axis labels is also provided in Supplementary Table 5. **E)** Protein-protein interaction network of all inferred transcriptional regulators identified by IPA analysis of the top 600 transcripts differentially expressed in fibropapillomatosis (RNA-seq). Network generated using the STRING database (v10.5, http://www.stringdb.org).

Supplementary Table 1. ChHV5 viral transcripts in fibropapillomatosis *de novo* transcriptome.

Transcript ID	Nearest gene(s) in ChHV5 genome	ChHV5 genome coordinates (bp) aligns to	Sequence
TRINITY_DN119680_c0_g1_i1	Inter-HP3/HP4 region transcript 1	5736 - 6061,<br>25246 - 24921,<br>5439 - 5609<br>and<br>25543 – 25373	GGCTCCGCCGCCGACCGGCCCCTCG<br>CCATCGGGCCCCGAAAGTTCACCCA<br>GGCCTGGGCCCTCGCGCCGATCTCC<br>ACCGTCCCAAACAAGAGGACCGCCG<br>GAAGACACCGGACCCTCTCGCGCGT<br>CGAACACTCCGCCTTCAAGTCACGC<br>CCTTCCACCTAGGGCTTTTTGGGTGC<br>AAACCGGTGTTTGGTACAGGGATTA<br>CAGCAGGCAATGGTTCGAAAGCCAC<br>TTTCCTGACCAGAGAGAAAGGATAG<br>TGGCGGGTGGCGTGGTACAGGCCGT<br>CTTGAGTGCCTCGCTGGGGCTCGCTG<br>AGTTTTTCGTCAATCCTGTACTCAAT<br>CCGCAAAACGTCCCCCCCAGTCCTTT<br>ACTGCTCCTTCTGCTCTCTCAAGCTA<br>GCAGCGCTTTCCGCCAAATCGTGACT<br>ACCCTGGACGGCCTGACTCAGGACA<br>ATCCTTTTTTCCCTGAAGGGCACGGG<br>TGGGAGCAGATGGCGCGCGTATGTA<br>CCGAGGCGCCCTCG
TRINITY_DN119680_c1_g1_i1	Inter-HP3/HP4 region transcript 2	6344 - 6038<br>and<br>24638 - 24941	GCTCTCTTGAGGCAAACCAAGTTTGT<br>GCGTGTTTTTATAAAAATATAATATC<br>CATCTTTATTATTAAACAAAAAACAT<br>AGCTTGGAATGAACCGACACGCTTC<br>GTGAGCGTATAGACATCGGAGTCAG<br>AGAGTGCTTAATGCGACTAGTCCTC<br>GCCTTCCTCCTCCGCCGGCGGCTGGG<br>CCCTCTGCAGACCTAGCCAGTTCACA<br>ACGTCCTCCCTGATTAGCTCAAAGG<br>GTACGTCTTCGGGGCCAAGGTGGCC<br>GTGGCCTGGCAACCCGTATTCTCGGC<br>GGATTCTCGAGGGCGCCTCGGTACA<br>TACCCG
TRINITY_DN119680_c2_g1_i1	HP3	4883 – 5268<br>and<br>26099 - 25714	GCGAGAGCGACGATTCGTCGAGCTC<br>CGAATCATCGCCACCGGATGTACGA<br>AAGAAACGCAAGAAACCCCCTAAAA<br>CCAGTAAGTCCCCTACAGGCCGTAA<br>AAAGCCCGACAGTCCACCCAAAAGA<br>AAGCTACCTCGCTTGGGTCGCTTACA<br>TAAAAGCCGATACGGGGGTTGGTGT<br>CGATACAGTCCTTCGACCTCCCCTTG<br>CTCTAGTGGAAGCGATGTCATATGT<br>GTGGGGAGTTTCGGAACCCCAATTG<br>AGGTACGAAGCGGCAGCAGCACGGA<br>CTCCCCGAATCCCTACTCTTCGCCCG<br>ACGATGGCCCGGCCAGATCGCCCGG<br>CGAAGAATCTTCGCCGCCTCGCACC<br>CCTCCTCCCCCCCAAGGGCCAAAAA<br>GGAAATCGGAG
TRINITY_DN158726_c0_g1_i1	F-LANA/HP2	4007 - 3706<br>and<br>26975 - 27264	TCTTCTTCTTCTTCTCCGGGGCCTCC<br>GCTCGGGCCGGGTTCGCTCGAGTGC<br>GGCGGTTCTGGCGGGGATCGGCCCG<br>GCCCTTCTCGTTCGGGACCCTCGCCT<br>CCAAAGCCGTCCGGTTCGTCGGACC<br>CGTCCTCGTCTTCTTCTCCCGGCCCG<br>GAGGAAGGGCCGGACCTCGGCCCCG<br>GCCTTCCTCCTCCCCCTGACCGCACG<br>GTTCTGGAACGGCCGCGGCTCGCTC<br>CCCGGGGTCGGCCGCGTCCCCGGCC<br>CCGGTCTCTGGTCCCGCTCCGGCTCT<br>CGCCTCGGCCTCGGCCTCGGCCCCG

Supplementary Table 2. Top 300 upregulated and top 300 downregulated transcripts (FP tumor Vs. control), called by DESeq2 and ranked by adjusted p-value.

Transcript ID	Homologous gene ID	baseMeanA	baseMeanB	baseMean	log2FoldChange	p-value	padj
Top 300 up regulated transcripts:							
TRINITY_DN115109_c0_g1	PITX2	15.16207731	674.3186078	476.5716487	5.408318086	4.16E-124	3.52E-119
TRINITY_DN152537_c0_g2	FNDC1	100.74653	1234.43917	894.3313779	3.585464781	1.28E-106	3.60E-102
TRINITY_DN150062_c3_g1	CPXM1	1250.421868	19392.77268	13950.06744	3.913301577	1.73E-106	3.67E-102
TRINITY_DN149008_c1_g1		1.67674517	3556.774513	2490.245183	9.868319768	4.91E-99	8.31E-95
TRINITY_DN152113_c0_g1	S1PR3	127.1112191	2190.795501	1571.690216	4.055605976	4.45E-96	6.28E-92
TRINITY_DN154746_c0_g1	NES	106.9684834	2322.777377	1658.034709	4.367452939	3.45E-87	2.92E-83
TRINITY_DN151118_c0_g1	LRRC15	77.57440444	3242.787918	2293.223864	5.245465306	7.49E-84	5.77E-80
TRINITY_DN152355_c2_g1	WNT5A	567.2422633	3865.086847	2875.733472	2.750682394	4.87E-81	3.44E-77
TRINITY_DN155020_c0_g3	NCAN	10.28444651	543.8493866	383.7799046	5.53878135	9.06E-76	5.91E-72
TRINITY_DN155213_c8_g2	NTN3	93.39961645	1438.009575	1034.626587	3.88337618	3.09E-71	1.75E-67
TRINITY_DN137131_c0_g1	HTRA3	165.2538232	2788.357926	2001.426695	4.005372107	1.09E-70	5.80E-67
TRINITY_DN142485_c0_g1	ITM2C	105.32933	1666.889661	1198.421562	3.913018802	7.43E-66	3.70E-62
TRINITY_DN155031_c2_g1	EMILIN2	201.9051473	2540.778575	1839.116547	3.596551677	3.45E-64	1.63E-60
TRINITY_DN146926_c7_g2	THBS2	782.8837475	9075.412115	6587.653605	3.483887953	1.32E-62	5.88E-59
TRINITY_DN151607_c2_g1	SEMA6D	159.2226308	751.0458324	573.4988719	2.222210926	2.23E-62	9.45E-59
TRINITY_DN154441_c1_g1	MMP23B	436.7540069	3271.57736	2421.130354	2.875765061	3.33E-62	1.34E-58
TRINITY_DN153547_c6_g1	LRRC3	27.29309152	897.2661064	636.2742019	4.87237527	1.93E-61	7.45E-58
TRINITY_DN155194_c2_g1		200.7107514	2426.765132	1758.948818	3.538058531	1.97E-60	7.24E-57
TRINITY_DN31376_c0_g1		135.3724735	3097.911297	2209.14965	4.405477143	3.08E-60	1.09E-56
TRINITY_DN152871_c6_g1	SLC16A5	306.2593513	1309.852424	1008.774502	2.084244499	3.61E-60	1.22E-56
TRINITY_DN142485_c1_g1	ITM2C	282.3820641	4020.691725	2899.198826	3.762642562	1.01E-58	3.28E-55
TRINITY_DN155502_c5_g1	PRSS35	55.67937427	681.4450031	493.7153144	3.549700774	3.36E-58	1.05E-54
TRINITY_DN154104_c4_g2		45.93662499	370.9825118	273.4687458	2.977443816	1.54E-56	4.66E-53
TRINITY_DN147506_c0_g1	SRPX	620.0545859	3887.679527	2907.392044	2.624155683	3.95E-55	1.16E-51
TRINITY_DN139338_c1_g1	COLGALT2	67.56994665	1155.281265	828.9678693	4.007096758	4.80E-54	1.35E-50
TRINITY_DN149614_c1_g1	KCTD12	148.8572038	1513.436318	1104.062584	3.293623603	1.10E-53	3.00E-50
TRINITY_DN31376_c0_g2		130.0010984	1715.77489	1240.042752	3.652292778	5.91E-53	1.56E-49
TRINITY_DN151691_c0_g1	MYL9	1659.701345	7904.760388	6031.242675	2.235624652	7.14E-53	1.83E-49
TRINITY_DN153313_c0_g2	CRABP2	6420.153179	40846.75421	30518.7739	2.643107969	1.23E-52	2.98E-49

TRINITY_DN145185_c1_g1	ROR2	260.5228808	1513.097079	1137.324819	2.515355789	2.04E-52	4.81E-49
TRINITY_DN90102_c0_g1	CTHRC1	102.4722621	4385.782371	3100.789338	5.194572135	9.39E-52	2.15E-48
TRINITY_DN155020_c0_g2	NCAN	8.709055749	338.2513803	239.3886829	5.080263994	2.72E-51	6.06E-48
TRINITY_DN137494_c1_g1	FGF13	19.25896873	628.0578645	445.4181957	4.832971495	1.85E-49	4.02E-46
TRINITY_DN123021_c0_g1	WIF1	14.87530674	435.1364961	309.0581393	4.702170455	6.23E-49	1.32E-45
TRINITY_DN145215_c1_g1	FGF14	40.21825015	705.9287898	506.2156279	4.026764943	2.38E-47	4.69E-44
TRINITY_DN149582_c2_g1	AVIL	216.4661909	2170.265625	1584.125795	3.26709721	4.47E-47	8.62E-44
TRINITY_DN145623_c0_g1	GULP1	78.09773765	476.3092058	356.8457653	2.582738204	1.12E-46	2.07E-43
TRINITY_DN149190_c2_g1		0.558915057	521.6256309	365.3056161	8.040803761	1.86E-46	3.36E-43
TRINITY_DN153689_c0_g1	IL13RA2	117.0779123	1762.031019	1268.545087	3.818349852	5.27E-46	8.92E-43
TRINITY_DN150062_c1_g1		13.28703792	268.7889862	192.1384017	4.205244989	2.64E-45	4.15E-42
TRINITY_DN151935_c0_g1	SFRP1	120.4707798	3157.260105	2246.223307	4.543498581	3.71E-45	5.71E-42
TRINITY_DN154104_c5_g1	AOX2P	37.51352192	408.1727174	296.9749587	3.382248834	1.10E-44	1.67E-41
TRINITY_DN154375_c2_g3	RNF165	18.54121151	236.9908853	171.4559832	3.592096727	1.36E-44	2.01E-41
TRINITY_DN153627_c8_g1	ADCYAP1R1	17.48843989	331.9015846	237.5776412	4.121847095	1.61E-44	2.35E-41
TRINITY_DN155893_c1_g1	DCHS2	13.70522136	411.5702492	292.2107408	4.704770722	4.23E-44	6.07E-41
TRINITY_DN145691_c1_g1	NT5C1A	25.59382122	249.5749749	182.3806288	3.215685466	4.72E-44	6.66E-41
TRINITY_DN147184_c2_g1	EGFLAM	624.3841233	6731.66996	4899.484209	3.363607401	5.77E-44	8.02E-41
TRINITY_DN155027_c0_g1		136.042203	735.3923203	555.5872851	2.406576416	7.63E-44	1.04E-40
TRINITY_DN153761_c1_g3	TPM2	815.1389348	9769.65297	7083.29876	3.504014715	7.70E-43	1.04E-39
TRINITY_DN153250_c7_g1	WNT10A	121.7216212	710.3215314	533.7415583	2.51681438	7.91E-43	1.05E-39
TRINITY_DN150997_c7_g1	FHOD3	67.26936789	333.2873502	253.4819555	2.286976655	2.43E-42	3.17E-39
TRINITY_DN138959_c0_g1	PLAUR	9.694542863	795.4910876	559.7521242	5.904108682	2.48E-42	3.19E-39
TRINITY_DN143220_c0_g1	BCAS1	49.31884051	479.132493	350.1883972	3.214312245	2.81E-42	3.55E-39
TRINITY_DN147043_c3_g1		6.741087999	156.9334203	111.8757206	4.367123393	2.85E-42	3.55E-39
TRINITY_DN152597_c0_g1	DAAM2	774.8412014	4026.154749	3050.760685	2.353634658	9.14E-42	1.09E-38
TRINITY_DN153895_c4_g1	ITGA10	186.0699683	944.4402656	716.9291764	2.321899931	1.13E-41	1.32E-38
TRINITY_DN152355_c4_g1	Wnt5A	447.1806474	2819.239546	2107.621877	2.624181851	1.28E-41	1.49E-38
TRINITY_DN151900_c3_g3		175.3865319	1961.568443	1425.71387	3.406095562	2.16E-40	2.44E-37
TRINITY_DN150126_c4_g5		54.57320117	414.8637126	306.7765592	2.886414741	8.04E-40	8.96E-37
TRINITY_DN147245_c2_g1	ZEB1	8.131992737	202.725885	144.3477173	4.467474277	1.72E-39	1.87E-36
TRINITY_DN144740_c0_g1	NXNL2	7.869570161	666.1965049	468.6984245	5.911689623	3.16E-39	3.39E-36
TRINITY_DN151833_c1_g1	NTRK3	22.29169343	387.984428	278.2766076	3.985191862	1.83E-38	1.94E-35
TRINITY_DN149008_c1_g2		1405.645813	1225192.453	858056.4106	7.8896094	2.08E-37	2.15E-34

TRINITY_DN152537_c0_g3	FNDC1	82.32615418	875.2926621	637.4027097	3.333073676	2.35E-37	2.39E-34
TRINITY_DN152248_c6_g2	ADAMTS4	96.76543203	1517.947208	1091.592675	3.847900787	2.60E-37	2.62E-34
TRINITY_DN147158_c5_g1		56.28426973	645.0619198	468.4286248	3.426694845	3.42E-37	3.41E-34
TRINITY_DN145623_c11_g1	GULP1	336.2458508	2010.893948	1508.499519	2.545239598	9.08E-37	8.94E-34
TRINITY_DN153431_c3_g1	ADCYAP1R1	23.97151991	291.4788684	211.2266639	3.504762868	1.00E-36	9.75E-34
TRINITY_DN140624_c6_g1	HEY2	330.0223481	3098.635068	2268.051252	3.162845345	2.18E-36	2.08E-33
TRINITY_DN155027_c1_g3	CYR61	144.7263289	799.0156844	602.7288777	2.436668666	2.53E-36	2.38E-33
TRINITY_DN149983_c3_g1	COL5A2	3128.720828	24791.19747	18292.45448	2.931728162	3.13E-36	2.91E-33
TRINITY_DN100901_c0_g1	SMOC2	117.1147642	1000.885714	735.7544289	3.031906864	2.33E-35	2.06E-32
TRINITY_DN148401_c0_g1	UCP2	9.309536615	330.3677496	234.0502857	4.853033822	2.75E-35	2.40E-32
TRINITY_DN155484_c5_g3		28.08975285	216.3063343	159.8413599	2.893345979	3.59E-35	3.04E-32
TRINITY_DN147113_c0_g1		3.304391208	165.7060974	116.9855856	5.268737715	5.37E-35	4.46E-32
TRINITY_DN147899_c1_g1	GCH1	44.58205358	304.9522562	226.8411954	2.728370656	9.72E-35	7.91E-32
TRINITY_DN154985_c1_g1		725.9710265	5609.175938	4144.214465	2.894475592	1.26E-34	1.02E-31
TRINITY_DN145042_c1_g1	GDNF	4.905463249	168.8849578	119.6911094	4.796288216	1.55E-34	1.24E-31
TRINITY_DN150006_c0_g1	ITGA8	45.59181385	849.4635544	608.3020322	4.055362787	3.00E-34	2.38E-31
TRINITY_DN149112_c11_g1	C1QTNF6	608.222541	5334.483683	3916.605341	3.065658036	3.24E-34	2.54E-31
TRINITY_DN88621_c0_g1		5.0877981	194.9881861	138.0180697	4.945868189	4.79E-34	3.72E-31
TRINITY_DN144766_c2_g1	NRSN2	0	373.471782	261.4302474	7.919472413	5.77E-34	4.40E-31
TRINITY_DN143526_c1_g1	SLC2A1	1394.391789	8700.329174	6508.547958	2.600664253	1.16E-33	8.70E-31
TRINITY_DN149491_c0_g1	GRIK5	5.921938956	252.6379767	178.6231654	5.030521051	1.51E-33	1.12E-30
TRINITY_DN149308_c6_g1	TIAM2	104.3212429	668.1548415	499.0047619	2.634212462	4.17E-33	3.05E-30
TRINITY_DN155829_c5_g2		69.52325091	477.3785323	355.0219479	2.733103998	6.37E-33	4.62E-30
TRINITY_DN61486_c0_g1		0.558915057	210.4602479	147.489848	6.913743506	9.82E-33	7.05E-30
TRINITY_DN145309_c0_g1	APCDD1	2213.821663	13213.57171	9913.646696	2.538571679	1.05E-32	7.45E-30
TRINITY_DN150573_c0_g2	CADPS	12.96709017	325.7250636	231.8976716	4.420845899	1.40E-32	9.80E-30
TRINITY_DN142164_c1_g1	GLIS1	15.43195838	468.3123874	332.4482587	4.643899291	2.79E-32	1.90E-29
TRINITY_DN153759_c2_g1	SIM1	61.10627304	259.0683682	199.6797397	2.069069877	5.04E-32	3.33E-29
TRINITY_DN152617_c9_g1	COL26A1	72.51467487	7116.833139	5003.5376	5.946531601	6.51E-32	4.27E-29
TRINITY_DN134367_c1_g1	BMPER	12.70904478	572.5718379	404.613	5.089644951	1.77E-31	1.13E-28
TRINITY_DN145836_c2_g1		8.048786286	138.2115964	99.16275336	3.945115113	1.89E-31	1.19E-28
TRINITY_DN150690_c2_g2		92.45372263	568.3196633	425.5598811	2.572999583	2.16E-31	1.35E-28
TRINITY_DN151517_c7_g2	HUNK	15.07044482	168.6401897	122.5692663	3.381344072	6.16E-31	3.81E-28

TRINITY_DN143526_c2_g1	SLC2A1	166.7555249	1121.766066	835.2629038	2.700389228	6.72E-31	4.12E-28
TRINITY_DN142092_c2_g1	ANXA6	709.1803814	3505.197453	2666.392331	2.275191556	6.77E-31	4.13E-28
TRINITY_DN136374_c0_g1	SNCAIP	104.8470556	729.4752752	542.0868093	2.745871901	6.91E-31	4.18E-28
TRINITY_DN148154_c7_g1	SUCNR1	56.42693646	390.5325876	290.3008923	2.742084174	9.29E-31	5.54E-28
TRINITY_DN151182_c7_g1		12.96174544	140.3736576	102.150084	3.317047722	1.77E-30	1.04E-27
TRINITY_DN61486_c1_g1		0	197.28915	138.102405	7.458220005	2.01E-30	1.18E-27
TRINITY_DN148701_c1_g3	PCDH9	2.771120012	121.0731107	85.58251346	4.979896229	2.07E-30	1.20E-27
TRINITY_DN141739_c1_g1	GREM2	264.5199466	2349.888283	1724.277782	3.075485528	2.35E-30	1.35E-27
TRINITY_DN153298_c0_g4	TRPA1	1.385560006	205.7605332	144.4480412	6.185716092	3.75E-30	2.13E-27
TRINITY_DN150342_c1_g1	SDC3	118.9360912	946.9311559	698.5326365	2.923525656	4.30E-30	2.43E-27
TRINITY_DN138679_c0_g1	SMOC2	310.2865942	2504.475162	1846.218591	2.944488212	4.65E-30	2.61E-27
TRINITY_DN139837_c0_g1	ATP6V1G2	13.14408029	139.8877179	101.8646266	3.302746143	5.28E-30	2.94E-27
TRINITY_DN155743_c0_g1	ARSI	86.08154027	2135.04166	1520.353624	4.386751074	7.38E-30	4.09E-27
TRINITY_DN130663_c1_g1	FHOD3	55.44575169	260.7977513	199.1921514	2.202705081	1.46E-29	8.04E-27
TRINITY_DN152248_c6_g1	ADAMTS4	43.76465256	550.3677414	398.3868148	3.529284525	1.71E-29	9.36E-27
TRINITY_DN143849_c0_g2	NCAM1	182.8954032	834.9660601	639.344863	2.166493363	2.22E-29	1.20E-26
TRINITY_DN137869_c0_g1	NUAK1	537.3596235	2483.896106	1899.935161	2.181187714	3.77E-29	2.00E-26
TRINITY_DN153674_c2_g2	CDC42EP4	139.4121681	935.8800993	696.9397199	2.69182049	3.81E-29	2.00E-26
TRINITY_DN133982_c0_g1	ADAM8	174.4927489	1604.592521	1175.562589	3.118609931	4.57E-29	2.39E-26
TRINITY_DN113854_c0_g1	NAT16	0.291185164	183.5343405	128.5613939	6.944430176	7.99E-29	4.10E-26
TRINITY_DN135033_c3_g1		9.637017689	122.5237501	88.6577304	3.529816509	9.47E-29	4.80E-26
TRINITY_DN149846_c1_g1	THY1	346.4394342	11412.34388	8092.572546	4.708766181	1.78E-28	8.76E-26
TRINITY_DN150402_c0_g1	NTN4	28.35659002	304.4977548	221.6554054	3.316289811	1.98E-28	9.68E-26
TRINITY_DN153944_c0_g1	PLPPR4	2.638814429	219.0710572	154.1413844	5.665042236	2.33E-28	1.13E-25
TRINITY_DN153248_c1_g1	THBS2	7.21683402	131.9369876	94.52094153	4.010094978	3.83E-28	1.85E-25
TRINITY_DN150942_c2_g1	FOXD1	22.41739583	219.8017926	160.5864736	3.204512581	4.50E-28	2.17E-25
TRINITY_DN145854_c3_g1	SP5	5.623295301	346.9381302	244.5436797	5.400685484	5.61E-28	2.67E-25
TRINITY_DN129789_c0_g1	TSHZ3	100.2945676	562.1861881	423.6187019	2.442593653	9.91E-28	4.66E-25
TRINITY_DN142006_c0_g1	PTK7	1113.74754	7009.194571	5240.560461	2.602193747	3.11E-27	1.44E-24
TRINITY_DN152502_c8_g1	PRSS35	84.29214941	1010.397289	732.5657468	3.454637431	4.10E-27	1.89E-24
TRINITY_DN153288_c6_g1		712.0788132	3319.654256	2537.381623	2.190782347	5.15E-27	2.36E-24
TRINITY_DN147673_c0_g1		3.132670989	98.58541877	69.94959444	4.59045702	6.13E-27	2.79E-24
TRINITY_DN153131_c1_g1		132.5275406	1791.563737	1293.852878	3.609380168	1.10E-26	4.98E-24
TRINITY_DN152368_c0_g1	OLFML2B	630.0055821	4723.960691	3495.774158	2.836446216	1.66E-26	7.40E-24

TRINITY_DN146486_c0_g3	CPT1C	208.6760777	1071.003657	812.3053835	2.321247923	1.86E-26	8.21E-24
TRINITY_DN136245_c0_g1	TBX15	235.0027976	1112.243379	849.0712044	2.209728991	2.59E-26	1.13E-23
TRINITY_DN147812_c3_g1	MYO1E	147.1676928	642.6243818	493.9873751	2.098043898	3.49E-26	1.51E-23
TRINITY_DN155206_c0_g1	MFAP2	380.748174	1771.188264	1354.056237	2.185062472	3.95E-26	1.69E-23
TRINITY_DN148651_c0_g1	EPHA8	12.81444804	469.5823522	332.551981	4.801062372	4.49E-26	1.91E-23
TRINITY_DN61378_c0_g1	C1QTNF5	76.02663005	832.2659543	605.394157	3.332660367	6.99E-26	2.93E-23
TRINITY_DN139643_c0_g1	RASD2	19.72721885	1084.24504	764.8896935	5.225147477	7.66E-26	3.20E-23
TRINITY_DN153431_c5_g1	TDRP	37.95264372	749.539292	536.0632975	4.080452172	8.52E-26	3.54E-23
TRINITY_DN145426_c1_g1	ATOH8	70.75430851	523.3492754	387.5707853	2.815735202	9.63E-26	3.98E-23
TRINITY_DN148395_c0_g1	EMILIN1	562.3921688	5765.118927	4204.3009	3.246513411	1.25E-25	5.12E-23
TRINITY_DN100100_c0_g1	CRABP1	79.71514638	1484.115899	1062.795673	3.998867796	2.06E-25	8.33E-23
TRINITY_DN151517_c7_g1	HUNK	38.38610986	296.7077593	219.2112645	2.86887152	2.33E-25	9.40E-23
TRINITY_DN152391_c9_g1	LURAP1	162.1676338	1491.553328	1092.737619	3.103484465	2.87E-25	1.15E-22
TRINITY_DN154278_c1_g1	PDGFRA	1066.76677	5639.671926	4267.800379	2.360550818	4.03E-25	1.60E-22
TRINITY_DN95919_c0_g1	CDH11	1370.411949	10903.79448	8043.779723	2.910569596	1.02E-24	3.92E-22
TRINITY_DN141709_c2_g1		22.00674572	182.2559484	134.1811876	2.975070934	1.04E-24	3.97E-22
TRINITY_DN153171_c3_g4	CNTNAP5	31.05912966	453.1094989	326.4943881	3.691426213	1.13E-24	4.29E-22
TRINITY_DN153360_c1_g1	GJB6	422.8729243	5369.972941	3885.842936	3.516903753	1.16E-24	4.39E-22
TRINITY_DN152706_c3_g2	LVRN	208.7557539	1194.714821	898.9271011	2.465490394	1.39E-24	5.23E-22
TRINITY_DN152038_c6_g1		24.28523021	261.9814147	190.6725594	3.294962119	1.72E-24	6.37E-22
TRINITY_DN148984_c0_g1	CXCL8	19.96626098	446.3078427	318.4053682	4.215028751	2.06E-24	7.51E-22
TRINITY_DN151534_c2_g2	SMAD6	27.98527972	118.038724	91.02269069	2.037681688	2.62E-24	9.52E-22
TRINITY_DN151989_c3_g1	TMEM173	79.58375934	341.7316751	263.0873003	2.075091468	3.44E-24	1.24E-21
TRINITY_DN57032_c0_g1	SDC2	2585.274554	17267.68906	12862.9647	2.675379003	3.55E-24	1.27E-21
TRINITY_DN149270_c1_g2	GJB6	1529.230899	9015.289137	6769.471665	2.507023671	3.79E-24	1.36E-21
TRINITY_DN144568_c7_g1	WNT2B	194.5984547	1163.421003	872.7742383	2.523900276	4.01E-24	1.43E-21
TRINITY_DN144335_c0_g1	PPP2R2B	133.2372001	628.0229753	479.5872427	2.201023214	5.17E-24	1.82E-21
TRINITY_DN150312_c3_g1	MELTF	10.53402845	128.6219806	93.19559499	3.454967848	8.33E-24	2.89E-21
TRINITY_DN145715_c1_g1		130.2241812	576.9248272	442.9146334	2.117929257	8.71E-24	3.01E-21
TRINITY_DN149353_c2_g1	GAL3ST4	407.6746143	1880.037358	1438.328535	2.170794414	9.99E-24	3.40E-21
TRINITY_DN153445_c0_g1	ITGB7	205.8694765	1685.313975	1241.480626	2.944794082	1.12E-23	3.79E-21
TRINITY_DN145764_c0_g1	MEF2D	300.3632807	1267.911836	977.6472692	2.048660567	1.97E-23	6.59E-21
TRINITY_DN154934_c8_g3		3.863306265	96.61707421	68.79094383	4.32002668	2.02E-23	6.73E-21
TRINITY_DN146359_c1_g2	SPON1	286.4670397	1642.347113	1235.583091	2.466314992	2.28E-23	7.58E-21

TRINITY_DN61486_c0_g2		0.291185164	125.7707181	88.12685822	6.406334399	2.62E-23	8.65E-21
TRINITY_DN154832_c2_g1	PRICKLE1	327.8473492	1979.297893	1483.86273	2.536539153	3.27E-23	1.07E-20
TRINITY_DN151050_c6_g1	SERTAD4	14.23095923	106.5644362	78.86439314	2.827485441	3.38E-23	1.11E-20
TRINITY_DN153288_c6_g2		285.80533	1192.612643	920.5704491	2.032735225	3.65E-23	1.18E-20
TRINITY_DN143932_c2_g2	MMP7	98.03249464	1000.85873	730.0108596	3.229509314	5.12E-23	1.63E-20
TRINITY_DN150690_c2_g1		10.52872113	78.82657659	58.33721995	2.815016949	6.24E-23	1.98E-20
TRINITY_DN153116_c6_g1	HLX	153.8259775	629.8386361	487.0348386	2.00435865	8.38E-23	2.64E-20
TRINITY_DN145854_c2_g2	SP5	1.801554847	168.8974963	118.7687139	5.589039629	1.53E-22	4.73E-20
TRINITY_DN139261_c1_g3		0	244.5308641	171.1716049	6.857176106	1.54E-22	4.74E-20
TRINITY_DN148137_c0_g1	RNF122	25.55975132	143.9710951	108.447692	2.430014733	1.56E-22	4.78E-20
TRINITY_DN104301_c0_g1	NREP	4154.293106	20278.48617	15441.22825	2.246364695	2.67E-22	8.01E-20
TRINITY_DN155321_c9_g1	COL4A1	319.0680315	2207.637491	1641.066653	2.715642181	3.07E-22	9.18E-20
TRINITY_DN153349_c0_g4	SH3BGRL2	2.157798507	208.3744972	146.5094876	5.61517498	3.20E-22	9.53E-20
TRINITY_DN153796_c9_g1	C2orf40	570.9397817	4155.163604	3079.896458	2.782984194	3.71E-22	1.10E-19
TRINITY_DN143052_c2_g1	RCN3	864.3592229	3806.787963	2924.059341	2.104725121	3.80E-22	1.12E-19
TRINITY_DN153608_c3_g1	CCBE1	7.759789713	160.7196169	114.8316687	4.072043296	3.81E-22	1.12E-19
TRINITY_DN151111_c4_g1	CPXM2	71.21573095	3168.234292	2239.128724	4.919595011	5.18E-22	1.51E-19
TRINITY_DN145233_c0_g1	GJC1	12.78783663	260.8941952	186.4622876	4.061509082	6.16E-22	1.79E-19
TRINITY_DN139181_c1_g1	STRADB	82.59158324	504.5731985	377.9787139	2.548136243	6.51E-22	1.88E-19
TRINITY_DN147874_c1_g1	MMP14	1917.864213	8273.174431	6366.581366	2.076048862	7.05E-22	2.03E-19
TRINITY_DN154869_c1_g3	KIAA1614	33.68955546	149.3666523	114.6635232	2.112504193	7.93E-22	2.28E-19
TRINITY_DN31879_c0_g1	NDNF	192.9059114	2015.224241	1468.528742	3.250068422	8.39E-22	2.40E-19
TRINITY_DN151886_c5_g1	TWIST2	513.0234803	2787.545429	2105.188844	2.391317278	1.01E-21	2.87E-19
TRINITY_DN130663_c0_g1	FHOD3	31.1710987	168.8633009	127.5556403	2.389250436	1.09E-21	3.09E-19
TRINITY_DN123759_c0_g1	TIMP1	422.0491014	9872.960787	7037.687281	4.223191588	1.10E-21	3.10E-19
TRINITY_DN150062_c0_g1	CPXM1	9.005510813	113.9424487	82.46136733	3.47630086	1.13E-21	3.18E-19
TRINITY_DN106641_c0_g2	CNFN	8.228002411	114.3439651	82.50917628	3.603906154	1.15E-21	3.21E-19
TRINITY_DN128303_c0_g1	NME4	82.09424222	410.0989543	311.6975407	2.277558264	1.27E-21	3.53E-19
TRINITY_DN104185_c0_g1	MFAP4	579.6905445	2741.103719	2092.679766	2.201179811	1.30E-21	3.61E-19
TRINITY_DN149173_c1_g1	NT5C1A	26.15277369	172.2342957	128.4098391	2.646953989	1.32E-21	3.66E-19
TRINITY_DN138110_c0_g1	GLIPR2	630.3680574	4379.978966	3255.095694	2.719266912	1.45E-21	4.00E-19
TRINITY_DN149111_c1_g2	CDH13	4.255845834	99.77124112	71.11662254	4.235077679	1.53E-21	4.21E-19
TRINITY_DN148386_c0_g2	CTSK	133.8141509	733.171417	553.3642372	2.400526555	1.57E-21	4.29E-19
TRINITY_DN141759_c0_g1	TMEM233	54.22323238	521.8710257	381.5766877	3.14913039	1.64E-21	4.45E-19

TRINITY_DN154992_c10_g1		45.24046277	377.175205	277.5947824	2.952788574	2.02E-21	5.44E-19
TRINITY_DN151779_c0_g1	WNT6	524.4735401	2490.212428	1900.490762	2.206823745	2.32E-21	6.19E-19
TRINITY_DN141336_c4_g1	RGL1	438.7673417	2753.234301	2058.894213	2.582529279	2.71E-21	7.23E-19
TRINITY_DN151712_c3_g2	ATP1A3	87.91505126	615.0773848	456.9286848	2.730900514	2.92E-21	7.72E-19
TRINITY_DN155357_c2_g4		20.06130311	196.5269075	143.5872262	3.159034216	3.12E-21	8.21E-19
TRINITY_DN144301_c0_g1	KMO	0	85.94911856	60.16438299	6.447911547	3.85E-21	1.01E-18
TRINITY_DN155538_c1_g2	WDR86	19.11081603	166.5078891	122.2887672	3.026019267	4.24E-21	1.11E-18
TRINITY_DN145803_c6_g1	PDE5A	1258.8926	6649.900165	5032.597895	2.35059416	4.95E-21	1.29E-18
TRINITY_DN154985_c2_g1	PDGFRB	937.5973423	6551.594021	4867.395018	2.724572095	5.52E-21	1.43E-18
TRINITY_DN147899_c1_g2	GCH1	29.32704791	211.7259788	157.0062995	2.764474448	6.00E-21	1.54E-18
TRINITY_DN144031_c0_g3	ENOX1	22.37044787	216.1995653	158.0508301	3.143720664	6.81E-21	1.74E-18
TRINITY_DN155359_c2_g3	SPOCK1	15.0823179	262.203502	188.0671468	3.849851347	7.30E-21	1.86E-18
TRINITY_DN129328_c1_g1		24.38217002	391.6937874	281.5003022	3.768774491	8.04E-21	2.04E-18
TRINITY_DN152105_c0_g1	PRPH	40.58547418	217.1036441	164.1481931	2.359260277	1.20E-20	3.00E-18
TRINITY_DN148867_c3_g1	TMEM235	1.164740655	80.77167611	56.88959548	5.209746301	2.22E-20	5.49E-18
TRINITY_DN146359_c1_g1	SPON1	635.6783539	4350.260418	3235.885798	2.694115093	2.47E-20	6.09E-18
TRINITY_DN138011_c0_g1		0.998365168	202.4597836	142.0213581	5.985320234	2.58E-20	6.29E-18
TRINITY_DN149983_c3_g2	COL5A2	1405.892506	13089.39048	9584.34109	3.094049513	2.58E-20	6.29E-18
TRINITY_DN148106_c4_g1	SEMA5B	135.803535	745.8369295	562.8269111	2.40196612	2.76E-20	6.71E-18
TRINITY_DN145471_c3_g1	SHANK1	6.327319164	104.5219517	75.06356193	3.778582263	3.39E-20	8.15E-18
TRINITY_DN154832_c1_g1	PRICKLE1	84.23195767	380.7393568	291.7871371	2.134243106	3.75E-20	9.00E-18
TRINITY_DN148984_c1_g1	CXCL8	54.3780631	713.451399	515.7293983	3.521673967	5.37E-20	1.26E-17
TRINITY_DN153400_c1_g3	SLC6A12	1.206343881	71.06947086	50.11053277	5.079839502	5.82E-20	1.36E-17
TRINITY_DN148867_c3_g2	TMEM235	0.332788389	85.40367488	59.88240893	5.963082529	6.05E-20	1.41E-17
TRINITY_DN152739_c3_g1	PCP4L1	9.257318757	82.25651296	60.3567547	3.013431024	6.79E-20	1.58E-17
TRINITY_DN154505_c6_g2	CNTNAP5	11.78197556	281.6468317	200.6873749	4.212647459	7.17E-20	1.66E-17
TRINITY_DN155893_c1_g4	DCHS2	3.080453131	74.81256219	53.29292947	4.179239327	8.42E-20	1.92E-17
TRINITY_DN149599_c2_g1	MARCKS	755.8710053	3127.865087	2416.266862	2.014861256	8.46E-20	1.93E-17
TRINITY_DN154104_c5_g3		9.195378987	97.77783424	71.20309767	3.261197021	9.83E-20	2.23E-17
TRINITY_DN155724_c0_g1	CEACAM1	90.18904633	511.611435	385.1847184	2.443509991	1.04E-19	2.34E-17
TRINITY_DN135996_c1_g1	BAG2	9.803430591	101.8477437	74.23444975	3.230661533	1.30E-19	2.92E-17
TRINITY_DN154869_c1_g1	KIAA1614	54.24471513	239.4442134	183.8843639	2.100163614	1.40E-19	3.13E-17
TRINITY_DN151184_c6_g1		3599.193043	166844.2953	117870.7646	4.885305523	1.88E-19	4.15E-17
TRINITY_DN148172_c1_g1	FBN3	105.1665254	747.4413264	554.7588861	2.740373268	2.26E-19	4.97E-17

TRINITY_DN155327_c0_g2	AQP11	61.24815087	471.4081491	348.3601496	2.840152547	2.73E-19	5.98E-17
TRINITY_DN155357_c2_g3	DACT3	4.23988647	75.66982951	54.2408466	3.835667954	2.80E-19	6.12E-17
TRINITY_DN146162_c4_g3	ADGRL1	3.901790764	105.2269101	74.82937433	4.291500739	2.86E-19	6.24E-17
TRINITY_DN134755_c0_g1	LEP	1.266095061	95.67475757	67.35215882	5.278176565	3.07E-19	6.68E-17
TRINITY_DN155206_c1_g1	MFAP2	273.6219282	1270.145671	971.1885481	2.170023691	3.81E-19	8.24E-17
TRINITY_DN147899_c0_g1		36.17034562	257.2906459	190.9545558	2.737508303	4.23E-19	9.10E-17
TRINITY_DN152001_c7_g2	ITGA5	1173.885135	6087.896524	4613.693107	2.320066172	5.45E-19	1.16E-16
TRINITY_DN150690_c0_g1	DCHS1	21.43717862	186.6982427	137.1199235	3.003047416	6.02E-19	1.28E-16
TRINITY_DN146601_c2_g1		2.924692277	70.84310283	50.46757966	4.187580954	8.18E-19	1.74E-16
TRINITY_DN150342_c0_g1	SDC3	64.88766872	398.6512765	298.5221941	2.545567984	8.40E-19	1.78E-16
TRINITY_DN146485_c3_g1	WFDC1	156.222464	641.4176575	495.8590994	2.004155776	1.02E-18	2.12E-16
TRINITY_DN135033_c2_g1		8.150140692	99.04781854	71.77851519	3.423642996	1.08E-18	2.23E-16
TRINITY_DN153889_c4_g1	LPAR4	188.5575777	809.1204963	622.9516207	2.061367875	1.10E-18	2.27E-16
TRINITY_DN150325_c0_g1	WBSCR17	75.8642286	504.3934957	375.8347155	2.649066478	1.19E-18	2.43E-16
TRINITY_DN142151_c7_g1	PRRX1	430.4324041	3010.394209	2236.405668	2.714558324	1.23E-18	2.50E-16
TRINITY_DN152480_c3_g1	LPAR4	52.30246607	271.5214733	205.7557712	2.318856645	1.33E-18	2.71E-16
TRINITY_DN155815_c5_g3	TEX2	28.34245351	149.7488739	113.3269478	2.348115963	1.88E-18	3.81E-16
TRINITY_DN153131_c0_g1	NGFR	108.4303557	1125.847921	820.6226516	3.217200688	2.37E-18	4.76E-16
TRINITY_DN143636_c2_g1	UGDH	1036.3246	5036.687875	3836.578893	2.230665708	2.39E-18	4.79E-16
TRINITY_DN137692_c0_g1	DOC2A	0.332788389	71.98100346	50.48653894	5.749201788	2.69E-18	5.37E-16
TRINITY_DN133980_c0_g1	SATB2	11.40980995	88.1765293	65.14651349	2.835760688	2.70E-18	5.39E-16
TRINITY_DN152927_c0_g1		0.868248175	76.95795973	54.13104627	5.277764074	2.94E-18	5.84E-16
TRINITY_DN138147_c1_g1	SOCS1	125.495708	1173.023138	858.7649089	3.084450911	3.09E-18	6.14E-16
TRINITY_DN150741_c0_g1		30.46618212	503.2775507	361.4341402	3.771575389	3.12E-18	6.17E-16
TRINITY_DN155690_c1_g1	TMEM91	4.882007978	295.1450727	208.0661533	5.0501683	3.57E-18	7.02E-16
TRINITY_DN154383_c0_g1		27.03070636	229.0604408	168.4515204	2.950571306	4.20E-18	8.17E-16
TRINITY_DN154444_c1_g1		14.69074589	88.93653359	66.66279728	2.518816821	4.24E-18	8.24E-16
TRINITY_DN155893_c1_g5	DCHS2	1.671437854	83.46085112	58.92402714	4.831965928	4.28E-18	8.29E-16
TRINITY_DN153116_c5_g2	ACTC1	6.431792296	175.2331605	124.59275	4.322405482	4.61E-18	8.86E-16
TRINITY_DN149008_c0_g1		92.29236672	89458.35423	62648.53567	6.314713692	5.29E-18	1.01E-15
TRINITY_DN145854_c2_g1	SP5	76.66003462	848.264786	616.7833606	3.292210796	5.37E-18	1.03E-15
TRINITY_DN153944_c0_g2	PLPPR4	3.05480927	122.4837937	86.65509837	4.682506663	9.59E-18	1.81E-15
TRINITY_DN61486_c1_g2		0	68.85757759	48.20030431	6.040446411	9.86E-18	1.85E-15
TRINITY_DN152262_c1_g1	DPF1	15.96745558	141.7740875	104.0320979	3.015225107	1.03E-17	1.93E-15

TRINITY_DN154383_c1_g1	CYS1	774.710667	3938.053912	2989.050938	2.288964763	1.08E-17	2.01E-15
TRINITY_DN154591_c5_g1	MLLT3	34.54629629	217.526091	162.6321526	2.570698503	1.13E-17	2.09E-15
TRINITY_DN153298_c0_g2	TRPA1	0.291185164	108.6877796	76.16880127	5.869165511	1.46E-17	2.70E-15
TRINITY_DN151712_c3_g1	ATP1A3	11.40980995	119.5820845	87.1304021	3.213845735	1.74E-17	3.18E-15
TRINITY_DN155893_c1_g2	DCHS2	0.558915057	59.3761423	41.73097412	5.294282398	1.77E-17	3.23E-15
TRINITY_DN153330_c3_g1		28.34901928	186.4292884	139.0052077	2.620205544	1.87E-17	3.40E-15
TRINITY_DN153053_c3_g1		99.66900729	465.9572491	356.0707766	2.178103827	1.95E-17	3.52E-15
TRINITY_DN149259_c5_g1	TGFBR2	122.7629228	755.1823616	565.45653	2.541014034	1.99E-17	3.60E-15
TRINITY_DN150183_c5_g1	KCNAB3	2.342321949	129.1651069	91.1182714	4.924284579	2.09E-17	3.76E-15
TRINITY_DN155512_c0_g5	TBX20	8.594860704	69.8710907	51.4882217	2.902549824	2.20E-17	3.96E-15
TRINITY_DN149740_c12_g1	CBLB	19.6847229	86.43520559	66.41006078	2.086015016	2.21E-17	3.96E-15
TRINITY_DN116051_c0_g1	PDE7B	196.5157365	981.0108694	745.6623295	2.263115091	2.44E-17	4.36E-15
TRINITY_DN148386_c0_g1	CTSK	1392.278721	6950.540976	5283.062299	2.263605065	2.66E-17	4.74E-15
TRINITY_DN149190_c1_g1		0	57.28604387	40.10023071	5.933449227	3.18E-17	5.62E-15
TRINITY_DN153400_c0_g3		1.718348396	71.58999073	50.62849803	4.647815008	3.78E-17	6.63E-15
TRINITY_DN142811_c1_g1	GAPDHS	67.99587948	573.0157634	421.5097982	2.941910538	4.14E-17	7.21E-15
TRINITY_DN146578_c1_g1		5.623257886	79.99711889	57.68496059	3.576240354	4.59E-17	7.97E-15
TRINITY_DN142321_c1_g1	ADCYAP1	60.57088809	411.46141	306.1942535	2.6718818	4.64E-17	8.04E-15
TRINITY_DN153360_c0_g1	GJB6	13.51093859	171.5492618	124.1377649	3.442375264	4.78E-17	8.27E-15
TRINITY_DN155480_c1_g1	EPHA5	51.87043704	231.3521709	177.5076508	2.103826199	4.88E-17	8.40E-15
TRINITY_DN145244_c5_g1	IRX1	50.17884972	294.8005772	221.4140589	2.481620661	6.59E-17	1.13E-14
TRINITY_DN144294_c9_g1		0.267729893	136.7329014	95.79334994	5.886608536	6.98E-17	1.19E-14
TRINITY_DN146754_c6_g1	ZIC1	17.07021905	382.7636035	273.0555881	4.077690506	7.84E-17	1.33E-14
TRINITY_DN153939_c2_g3	LMOD1	50.32596003	409.4969162	301.7456293	2.892798747	7.87E-17	1.33E-14
TRINITY_DN151297_c2_g1	ACTA2	91.19426817	846.2734768	619.7497142	3.060475768	8.18E-17	1.38E-14
TRINITY_DN153871_c1_g2		1.159433339	85.65881077	60.30899754	5.082159037	9.00E-17	1.51E-14
TRINITY_DN141535_c1_g4	ADGRL1	10.01890521	101.4178408	73.99816015	3.150099073	1.12E-16	1.87E-14
TRINITY_DN155512_c0_g4	TBX20	8.249231677	125.0433163	90.00509094	3.639902218	1.39E-16	2.29E-14
TRINITY_DN146691_c5_g3	SH2B2	21.92572786	111.766967	84.81459524	2.289007973	1.41E-16	2.31E-14
TRINITY_DN152081_c4_g2		108.1146729	535.57158	407.3345079	2.250992051	1.57E-16	2.57E-14
TRINITY_DN141599_c0_g1		0.267729893	66.6697254	46.74912675	5.567104706	1.80E-16	2.93E-14
TRINITY_DN155347_c6_g2	LRRC27	27.78353846	185.2408427	138.0036514	2.643714365	1.88E-16	3.05E-14
TRINITY_DN144472_c0_g2	COQ8A	5.440923035	126.2442287	90.003237	4.087444016	1.94E-16	3.14E-14
TRINITY_DN153687_c5_g1		140.5551525	889.5277166	664.8359474	2.57116725	1.95E-16	3.15E-14

Top 300 down regulated transcripts:

TRINITY_DN153673_c8_g1	SULT1A4	3361.078099	17.05513266	1020.262023	-7.345444834	3.13E-110	1.33E-105
TRINITY_DN144332_c0_g1	KRT7	489.3138418	5.274906286	150.4865869	-6.304233307	4.01E-89	4.85E-85
TRINITY_DN152568_c4_g1		2242.017127	39.00181324	699.9064072	-5.689424304	6.50E-88	6.88E-84
TRINITY_DN132030_c1_g1		4503.01923	10.87163802	1358.515916	-8.202920501	8.38E-88	7.89E-84
TRINITY_DN145658_c0_g1	F10	828.3863768	130.8275742	340.0952149	-2.644119879	1.36E-73	8.21E-70
TRINITY_DN154564_c5_g1		353.0373253	1.233382657	106.7745655	-7.361229734	8.46E-53	2.11E-49
TRINITY_DN142587_c1_g1	KRT24	27258.70804	666.2879435	8644.013973	-5.132611445	9.79E-48	1.97E-44
TRINITY_DN145449_c0_g1		389.2249752	0.851012042	117.363201	-7.739533708	7.85E-47	1.48E-43
TRINITY_DN154801_c10_g1	LRP4	2742.861461	627.7573947	1262.288615	-2.111921269	2.50E-46	4.36E-43
TRINITY_DN147614_c0_g1		212086.6148	169.0084116	63744.29034	-8.888555311	1.66E-45	2.71E-42
TRINITY_DN124277_c0_g1	SOD3	429.6546453	16.46302792	140.4205131	-4.540435212	6.79E-42	8.22E-39
TRINITY_DN150292_c1_g4		2390.293406	64.40953036	762.1746929	-4.954804443	5.04E-38	5.27E-35
TRINITY_DN145583_c0_g1		698315.6558	32509.05736	232251.0369	-4.257603472	1.67E-36	1.60E-33
TRINITY_DN135765_c2_g1	SOX5	144.0305324	16.82800186	54.98876103	-3.037230744	1.91E-35	1.72E-32
TRINITY_DN150292_c1_g3		3256.222447	24.3099198	993.8836779	-6.418531399	3.07E-35	2.65E-32
TRINITY_DN152503_c4_g3	SNX29	286.4950677	35.02190461	110.4638535	-2.977343572	3.36E-35	2.88E-32
TRINITY_DN152568_c1_g2	PPRC1	2843.262735	627.3131946	1292.098057	-2.157895874	5.30E-34	4.08E-31
TRINITY_DN147686_c0_g1	ASPN	52518.53629	1571.42692	16855.55973	-4.798162235	7.50E-34	5.67E-31
TRINITY_DN154858_c4_g1	GAS2	711.8023527	91.03712859	277.2666958	-2.90790248	1.36E-32	9.62E-30
TRINITY_DN148274_c3_g1	FGFR3	7195.592927	947.5867694	2821.988617	-2.868496125	1.79E-32	1.24E-29
TRINITY_DN146062_c8_g2	ENPP6	125.1317814	13.83060857	47.22096041	-3.114142176	4.63E-32	3.09E-29
TRINITY_DN150292_c1_g1		8280.943584	72.62320761	2535.119321	-6.167014963	8.48E-31	5.10E-28
TRINITY_DN135684_c2_g1		259210.2751	21256.67583	92642.75562	-3.490063417	9.68E-29	4.88E-26
TRINITY_DN152084_c3_g1	BMP5	404.1193574	20.04769903	135.2691965	-4.140312964	9.97E-29	5.00E-26
TRINITY_DN149889_c8_g1	SHH	731.4318365	74.53919976	271.6069908	-3.20387862	1.67E-28	8.26E-26
TRINITY_DN135684_c0_g1		64922.18871	4326.696205	22505.34396	-3.752541078	1.37E-27	6.35E-25
TRINITY_DN152568_c1_g1	CCER2	245.3498637	5.802440049	77.66666715	-4.968089339	2.70E-26	1.17E-23
TRINITY_DN67015_c0_g1		200.7526655	44.18629317	91.15620487	-2.15552312	5.32E-26	2.25E-23
TRINITY_DN148749_c8_g4		114.1870654	7.736837658	39.67190598	-3.729477587	6.71E-26	2.83E-23
TRINITY_DN143860_c3_g1	KRT5	97.76994823	0.310092905	29.5480495	-6.622905112	3.66E-25	1.46E-22
TRINITY_DN141347_c0_g1	WIPF3	302.9328987	28.46559727	110.8057877	-3.297332509	5.05E-25	1.98E-22
TRINITY_DN151129_c4_g1	DBNDD1	502.8612001	115.5390696	231.7357087	-2.092623165	1.30E-24	4.89E-22

TRINITY_DN155086_c3_g1	CCER2	1917.353535	22.07855888	590.6610517	-5.735864095	1.68E-24	6.28E-22
TRINITY_DN146348_c1_g1	EDAR	345.5811957	11.87355299	111.9858458	-4.540771462	1.88E-24	6.88E-22
TRINITY_DN143947_c0_g1	PTCH1	2385.603266	515.7156041	1076.681903	-2.176218157	4.61E-24	1.63E-21
TRINITY_DN154564_c7_g1		283.8365475	1.761940873	86.38432286	-6.254812522	6.83E-24	2.38E-21
TRINITY_DN139535_c0_g1	FAM134B	1540.178212	104.2694624	535.0420872	-3.707629441	9.42E-24	3.23E-21
TRINITY_DN143506_c0_g2	NELL2	345.0253388	71.85840772	153.808487	-2.230475854	2.89E-23	9.52E-21
TRINITY_DN151223_c8_g4		129.2525054	0.412555323	39.06454035	-6.544488122	3.41E-23	1.11E-20
TRINITY_DN148977_c9_g1		82.9251521	0.436224358	25.18290268	-6.178237327	6.61E-23	2.09E-20
TRINITY_DN146681_c5_g1		113.3069067	8.84040644	40.18035653	-3.532793571	1.59E-22	4.86E-20
TRINITY_DN148274_c2_g1		151.5306151	8.471481025	51.38922126	-3.918618096	1.63E-21	4.45E-19
TRINITY_DN108670_c0_g1		179.7660818	0.124692446	54.01710924	-7.028335521	1.98E-21	5.37E-19
TRINITY_DN125052_c0_g1		184.1572707	2.723766989	57.15381812	-5.364488172	2.03E-21	5.46E-19
TRINITY_DN146196_c3_g1	APCDD1L	2129.983867	445.5126463	950.8540126	-2.216144236	4.64E-21	1.21E-18
TRINITY_DN146458_c2_g1		68.06806814	0.140548073	20.51880409	-6.414653448	5.42E-21	1.40E-18
TRINITY_DN135684_c0_g2		115220.8373	8319.885565	40390.17109	-3.603665211	6.80E-21	1.74E-18
TRINITY_DN149994_c3_g2	KRT5	266.2062962	46.75890277	112.5931208	-2.450990799	9.63E-21	2.43E-18
TRINITY_DN146196_c4_g1	APCDD1L	1232.253021	280.046469	565.7084345	-2.100457726	3.39E-20	8.15E-18
TRINITY_DN152106_c5_g1	ARHGEF37	941.1276762	181.5833781	409.4466676	-2.323688536	4.94E-20	1.18E-17
TRINITY_DN150758_c9_g1		544.0183177	85.10283311	222.7774785	-2.606181292	7.00E-20	1.62E-17
TRINITY_DN143860_c0_g16	KRT1	25257.51192	48.44089987	7611.162205	-6.946661175	7.28E-20	1.68E-17
TRINITY_DN150784_c7_g1	HSPA12A	178.6727636	14.11830443	63.48464217	-3.485612332	7.86E-20	1.80E-17
TRINITY_DN143860_c0_g10	KRT1	183.0507293	0	54.91521879	-7.082574132	8.98E-20	2.04E-17
TRINITY_DN151989_c5_g2		190.1306793	20.19400404	71.17500661	-3.103717818	1.30E-19	2.92E-17
TRINITY_DN131524_c0_g1		8822.214316	35.36482494	2671.419672	-6.424887493	3.17E-19	6.87E-17
TRINITY_DN151756_c5_g2	ABI3BP	3742.289339	869.0299759	1731.007785	-2.068539805	8.60E-19	1.81E-16
TRINITY_DN139172_c0_g1	SOX14	115.4459929	1.042157065	35.36330781	-5.673810072	9.12E-19	1.91E-16
TRINITY_DN140837_c2_g1	PLA2G4C	568.3062778	8.675978607	176.5650684	-5.27338171	9.31E-19	1.95E-16
TRINITY_DN152936_c5_g1	BMP3	8293.087185	1048.94542	3222.18795	-2.87559614	1.94E-18	3.92E-16
TRINITY_DN142587_c0_g1		86.26851738	0.718651893	26.38361154	-5.65246675	3.80E-18	7.46E-16
TRINITY_DN149558_c9_g1	UOX	71183.26609	6162.643574	25668.83033	-3.352207283	4.51E-18	8.70E-16
TRINITY_DN140768_c1_g1	GPX3	285.6549718	43.96161756	116.4696239	-2.619380349	6.69E-18	1.27E-15
TRINITY_DN150784_c7_g3	HSPA12A	250.8312696	23.46882292	91.67755692	-3.259729298	8.11E-18	1.53E-15
TRINITY_DN149809_c5_g1		88.96989922	6.601120458	31.31175409	-3.518326601	1.15E-17	2.13E-15
TRINITY_DN144268_c2_g1		83.73480372	8.387362259	30.9915947	-3.155399619	1.37E-17	2.53E-15

TRINITY_DN151484_c8_g2	GPNMB	1106.940328	218.1817398	484.8093163	-2.286735167	1.60E-17	2.93E-15
TRINITY_DN149844_c0_g1	WDYHV1	318.5930369	5.375390643	99.34068453	-5.114320249	1.74E-17	3.18E-15
TRINITY_DN147029_c10_g1		88.81847813	19.93781411	40.60201332	-2.104293021	1.91E-17	3.46E-15
TRINITY_DN139913_c0_g1	SLC6A9	580.0756345	109.9694816	251.0013275	-2.339753811	2.69E-17	4.79E-15
TRINITY_DN150818_c0_g1		66.67271139	0	20.00181342	-6.393661095	3.02E-17	5.34E-15
TRINITY_DN146463_c0_g1	MYOM1	344.0919056	54.06275841	141.0715026	-2.585178524	6.74E-17	1.15E-14
TRINITY_DN148274_c4_g1	FGFR3	241.0557579	16.47479019	83.84908049	-3.621118043	9.51E-17	1.59E-14
TRINITY_DN144739_c0_g1	ADGRD1	84.94922532	1.225018088	26.34228026	-5.202502829	1.04E-16	1.75E-14
TRINITY_DN105839_c0_g4	MT1A	1359.545942	286.2932505	608.2690579	-2.196050736	1.06E-16	1.77E-14
TRINITY_DN132030_c0_g1		91.36195111	1.923268424	28.75487323	-4.861282882	1.41E-16	2.31E-14
TRINITY_DN149558_c13_g2	UOX	1210.888443	4.346434755	366.3090372	-6.230013878	2.22E-16	3.56E-14
TRINITY_DN155230_c3_g2	PGLYRP3	510.5031169	77.79587192	207.6080454	-2.619673312	3.12E-16	4.93E-14
TRINITY_DN154919_c0_g1	NCKAP5	517.8138531	94.46541452	221.4699461	-2.384388932	3.55E-16	5.56E-14
TRINITY_DN147519_c5_g1		120.3148693	10.7685228	43.63242676	-3.293531648	9.41E-16	1.42E-13
TRINITY_DN136233_c0_g2	KCNF1	43.12605293	0.295638295	13.14476269	-5.501098826	1.09E-15	1.62E-13
TRINITY_DN68681_c0_g1	NTS	109.5215628	0	32.85646885	-6.368591851	2.18E-15	3.14E-13
TRINITY_DN155492_c1_g3		197.8544091	6.592869781	63.97133159	-4.371680576	2.88E-15	4.08E-13
TRINITY_DN146062_c8_g1	ENPP6	601.9665236	53.51789435	218.0524831	-3.280661292	6.36E-15	8.62E-13
TRINITY_DN144341_c4_g1	EPDR1	591.7443078	43.73309395	208.1364581	-3.499238479	8.17E-15	1.09E-12
TRINITY_DN148689_c0_g1	CRYM	195.202947	33.36072623	81.91339245	-2.461749136	8.57E-15	1.14E-12
TRINITY_DN150555_c2_g2	PTCH2	2406.003114	218.9560852	875.0701937	-3.24511783	2.47E-14	3.07E-12
TRINITY_DN144161_c0_g1	CFAP47	90.18645458	8.131663295	32.74810068	-3.248232079	2.81E-14	3.45E-12
TRINITY_DN147686_c1_g1	EDAA8	3253.886779	22.29260619	991.7708581	-5.647975144	2.90E-14	3.55E-12
TRINITY_DN155234_c3_g1		499.1704778	41.41351036	178.7406006	-3.350972917	3.97E-14	4.79E-12
TRINITY_DN149889_c5_g1	SHH	229.0857254	12.63814592	77.57241976	-3.808673716	5.65E-14	6.73E-12
TRINITY_DN151989_c5_g1	daf-36-like	2091.180718	241.7657131	796.5902146	-2.950035557	6.10E-14	7.23E-12
TRINITY_DN142827_c0_g1		158.8008996	6.149573398	51.94497127	-4.159748517	8.92E-14	1.03E-11
TRINITY_DN154338_c4_g2	KCNQ3	71.24716011	0.746480781	21.89668458	-5.208728286	9.50E-14	1.10E-11
TRINITY_DN151223_c8_g1		87.81632918	1.460873721	27.36751036	-4.902440422	1.03E-13	1.19E-11
TRINITY_DN147225_c4_g1		182.6726893	12.36096176	63.45448004	-3.567059042	1.60E-13	1.82E-11
TRINITY_DN145442_c1_g1		117.3817884	21.63005917	50.35557794	-2.358443047	2.68E-13	2.94E-11
TRINITY_DN152281_c2_g2		49.81792955	0.124692446	15.03266358	-5.57796144	3.57E-13	3.82E-11
TRINITY_DN155492_c1_g1		396.2010391	22.25646005	134.4398338	-3.760779768	5.09E-13	5.31E-11
TRINITY_DN142413_c0_g1	LUZP2	84.73517041	1.515429326	26.48135165	-4.766089144	7.80E-13	7.92E-11

TRINITY_DN144413_c2_g1		145.0412861	15.73837942	54.52925141	-3.009239248	8.21E-13	8.31E-11
TRINITY_DN155492_c1_g2		374.6723059	18.05852009	125.0426559	-3.912028736	8.35E-13	8.44E-11
TRINITY_DN155769_c2_g2	ANK1	443.0829671	84.65371699	192.182492	-2.302749846	1.44E-12	1.40E-10
TRINITY_DN148657_c0_g1	SEMA3A	364.9655037	55.57130338	148.3895635	-2.59283345	1.60E-12	1.55E-10
TRINITY_DN152208_c1_g1	SELP	133.8737782	20.90077051	54.79267283	-2.556247468	2.55E-12	2.41E-10
TRINITY_DN154106_c0_g1	TUBB3	528.4951468	123.982966	245.3366203	-2.030946876	3.93E-12	3.61E-10
TRINITY_DN160565_c0_g1		63.07283267	2.663088367	20.78601166	-3.991010172	4.23E-12	3.87E-10
TRINITY_DN148422_c4_g1	KLHL3	333.3708885	68.82153864	148.1863436	-2.199533437	4.40E-12	4.01E-10
TRINITY_DN151063_c0_g2	SOX10	149.6781009	13.92497001	54.65090927	-3.181949281	4.58E-12	4.16E-10
TRINITY_DN151484_c7_g1	GPNMB	1297.391698	249.4945639	563.8637042	-2.291043778	4.74E-12	4.31E-10
TRINITY_DN152075_c1_g1	ZNF436	58.27257403	6.497476877	22.03000602	-2.966345702	6.48E-12	5.79E-10
TRINITY_DN155922_c0_g1	CYP2R1	82.50622561	15.47078035	35.58141393	-2.31476532	7.53E-12	6.66E-10
TRINITY_DN149996_c8_g1	MUC1	3775.726117	854.4912118	1730.861683	-2.076974157	9.53E-12	8.34E-10
TRINITY_DN133385_c0_g1		33.25537525	0	9.976612576	-5.412672242	9.64E-12	8.43E-10
TRINITY_DN153458_c6_g1	C4orf22	515.9546255	117.6849231	237.1658338	-2.06674615	1.07E-11	9.31E-10
TRINITY_DN149994_c4_g1	KRT5	122.0299119	27.10355344	55.58146098	-2.099129482	1.19E-11	1.02E-09
TRINITY_DN146194_c1_g1		11260.49177	1465.154986	4403.75602	-2.774362799	1.32E-11	1.13E-09
TRINITY_DN139091_c1_g2		29.16601715	0.265240519	8.935473509	-4.902798578	1.54E-11	1.29E-09
TRINITY_DN39800_c0_g1	KRT24	60.4272654	0.464683082	18.45345778	-5.127347475	1.56E-11	1.31E-09
TRINITY_DN139365_c0_g1	MUC1	958.3532646	187.2195004	418.5596297	-2.265418754	1.95E-11	1.61E-09
TRINITY_DN152954_c6_g2		191.6233126	16.27394688	68.87875658	-3.265066283	2.33E-11	1.90E-09
TRINITY_DN146348_c2_g2	EDAR	295.6042369	13.33047679	98.01260482	-3.922466573	2.46E-11	2.01E-09
TRINITY_DN155016_c3_g1	EDAA8	18592.65644	312.5237974	5796.563591	-4.760756058	2.51E-11	2.04E-09
TRINITY_DN138325_c0_g3	CALCB	33.82490494	0	10.14747148	-5.341371258	3.30E-11	2.64E-09
TRINITY_DN83076_c0_g1		92.05425155	9.265864327	34.10238049	-3.068803741	3.50E-11	2.78E-09
TRINITY_DN154564_c6_g1		88.48776609	12.26920393	35.13477258	-2.687090839	3.96E-11	3.11E-09
TRINITY_DN136710_c0_g3	WIPF3	45.38390997	3.313487444	15.9346142	-3.41423549	3.97E-11	3.11E-09
TRINITY_DN135765_c1_g1	SOX5	39.46018558	2.006376778	13.24251942	-3.7716064	4.01E-11	3.14E-09
TRINITY_DN152936_c4_g1		55.2098657	6.818595371	21.33597647	-2.831585465	4.08E-11	3.19E-09
TRINITY_DN141126_c0_g1	PPP4R4	108.0630369	21.78195537	47.66627982	-2.226125893	4.83E-11	3.73E-09
TRINITY_DN150555_c2_g1	PTCH2	5694.243743	679.778872	2184.118333	-2.867408527	4.83E-11	3.73E-09
TRINITY_DN137359_c0_g1	FOXI3	421.4298159	5.463304075	130.2532576	-4.890485557	4.97E-11	3.83E-09
TRINITY_DN111076_c0_g1		29.160635	0	8.748190501	-5.243129438	5.44E-11	4.16E-09
TRINITY_DN151063_c0_g3	SOX10	126.1504457	11.64422732	45.99609282	-3.166022641	5.82E-11	4.43E-09

TRINITY_DN147772_c1_g1	KIAA1456	35.50165011	1.929327271	12.00102412	-3.719807982	5.94E-11	4.51E-09
TRINITY_DN152220_c5_g1		146.505347	27.20932941	62.9981347	-2.317474455	7.15E-11	5.35E-09
TRINITY_DN153068_c2_g3	SCN3B	89.24731361	17.1405203	38.7725583	-2.289318204	7.65E-11	5.68E-09
TRINITY_DN154414_c7_g1		144.4605257	22.2958376	58.94524403	-2.552718965	8.64E-11	6.38E-09
TRINITY_DN151032_c5_g1	GFPT2	1151.026746	216.4848963	496.8474512	-2.308165182	9.59E-11	7.01E-09
TRINITY_DN154292_c0_g2	FAM107A	93.94366935	1.438605704	29.1901248	-4.709537477	1.02E-10	7.41E-09
TRINITY_DN135791_c0_g1	GPC6	33.66086771	0.12877632	10.18840374	-5.073963376	1.03E-10	7.48E-09
TRINITY_DN138325_c0_g6	CALCB	404.5236341	21.88311234	136.6752689	-3.717131343	1.04E-10	7.56E-09
TRINITY_DN149377_c1_g4	PLXNA4	74.93092193	8.955895306	28.74840329	-2.865901209	1.05E-10	7.62E-09
TRINITY_DN140443_c1_g1	TFAP2B	96.45305502	14.59385953	39.15161818	-2.580341707	1.22E-10	8.75E-09
TRINITY_DN116096_c1_g1	GLI3	50.98949789	6.114716799	19.57715113	-2.856008078	1.50E-10	1.06E-08
TRINITY_DN134272_c0_g1	CHRDL1	1325.406722	13.33894503	406.9592782	-4.982332978	1.61E-10	1.13E-08
TRINITY_DN147749_c2_g1	NAA40	31.0334858	0	9.31004574	-5.185471868	1.85E-10	1.29E-08
TRINITY_DN150686_c7_g2	ALK	243.6353333	35.55630613	97.9800143	-2.614054718	1.93E-10	1.33E-08
TRINITY_DN140443_c2_g1	TFAP2B	492.6841958	51.64077121	183.9537986	-3.00469296	2.03E-10	1.40E-08
TRINITY_DN148922_c1_g1	LIN7A	574.0167709	108.5841125	248.21391	-2.295318665	3.26E-10	2.17E-08
TRINITY_DN146137_c0_g2		119.9427411	27.37634885	55.14626653	-2.043892164	4.01E-10	2.66E-08
TRINITY_DN142620_c0_g1	SBSPON	277.3432528	14.60113371	93.42376945	-3.712438849	4.04E-10	2.67E-08
TRINITY_DN144121_c4_g1	PTGIS	722.1805656	78.3095148	271.47083	-2.957897706	4.57E-10	2.98E-08
TRINITY_DN148009_c1_g1	NEBL	250.8630761	43.29104739	105.562656	-2.406243339	6.30E-10	4.03E-08
TRINITY_DN147686_c3_g1		16986.21302	3421.543375	7490.944268	-2.211670812	6.75E-10	4.31E-08
TRINITY_DN149419_c6_g1		58.78866482	2.000678818	19.03707462	-4.058421785	6.76E-10	4.31E-08
TRINITY_DN152789_c9_g1		363.7401424	47.54085919	142.4006441	-2.73569172	7.39E-10	4.67E-08
TRINITY_DN150295_c8_g1	THAP6	37.73645503	0.42164422	11.61608746	-4.677257839	7.73E-10	4.87E-08
TRINITY_DN141729_c6_g1	KRT1	44.4323431	0.309355432	13.54625173	-4.870835499	8.51E-10	5.32E-08
TRINITY_DN148755_c2_g1	SLC8A1	27.7216361	0.283779003	8.515136132	-4.66064974	8.63E-10	5.38E-08
TRINITY_DN131870_c0_g1	RGR	257.5460197	55.36695095	116.0206716	-2.127908757	9.64E-10	5.97E-08
TRINITY_DN148830_c5_g1		276.4895933	59.66050249	124.7092297	-2.120635668	1.01E-09	6.23E-08
TRINITY_DN137741_c0_g1	PDGFC	782.9787865	117.934576	317.4478392	-2.566787464	1.05E-09	6.43E-08
TRINITY_DN144973_c4_g1		79.76012182	1.520319857	24.99226045	-4.467723664	1.09E-09	6.63E-08
TRINITY_DN153642_c0_g3	SCUBE1	980.1166705	111.2963061	371.9424154	-2.894313819	1.13E-09	6.84E-08
TRINITY_DN143896_c11_g2		85.02479462	7.154665331	30.51570412	-3.214596446	1.21E-09	7.31E-08
TRINITY_DN153642_c0_g4	SCUBE1	576.317224	68.26088703	220.6777881	-2.844573931	1.28E-09	7.73E-08
TRINITY_DN139091_c0_g1		26.9975666	0.249384891	8.273839403	-4.635504253	1.36E-09	8.11E-08

TRINITY_DN143860_c0_g13	KRT5	3840.632667	7.305345059	1157.303542	-5.232756434	1.40E-09	8.36E-08
TRINITY_DN154292_c0_g1	FAM107A	59.91127526	0.310130894	18.1904742	-4.937169447	1.52E-09	8.99E-08
TRINITY_DN138186_c0_g1		38.75705443	3.227407455	13.88630155	-3.201942502	1.71E-09	1.00E-07
TRINITY_DN155922_c0_g2	CYP2R1	87.93731449	16.05950531	37.62284807	-2.329378112	2.17E-09	1.25E-07
TRINITY_DN155362_c2_g1	KLK15	895.5711791	183.2035227	396.9138196	-2.186127742	2.48E-09	1.41E-07
TRINITY_DN137250_c0_g1	ARHGAP24	72.43854959	8.722104633	27.83703812	-2.821137262	2.51E-09	1.43E-07
TRINITY_DN147473_c2_g1		97.79526377	16.0764998	40.59212899	-2.450648304	2.57E-09	1.46E-07
TRINITY_DN147174_c0_g2	NTM	158.5741306	13.84251353	57.26199864	-3.164814123	2.77E-09	1.56E-07
TRINITY_DN146574_c0_g2	SUSD4	73.2704012	4.720104347	25.2851934	-3.46272314	3.16E-09	1.76E-07
TRINITY_DN151093_c0_g3		128.6914766	19.34910799	52.15181857	-2.561233984	3.16E-09	1.76E-07
TRINITY_DN145145_c5_g2	CLEC2B	241.9578283	24.8289112	89.96758632	-2.994853269	3.21E-09	1.79E-07
TRINITY_DN153953_c0_g3	COL10A1	93.30557929	5.394268407	31.76766167	-3.555913377	3.40E-09	1.88E-07
TRINITY_DN141729_c5_g1	KRT1	37.72317383	0	11.31695215	-4.985170026	3.65E-09	2.01E-07
TRINITY_DN128053_c0_g1		44.9467607	6.818694133	18.2571141	-2.535380593	4.12E-09	2.23E-07
TRINITY_DN153284_c6_g1	CHORDC1	32.03622815	1.894480045	10.93700448	-3.494711155	4.18E-09	2.27E-07
TRINITY_DN134297_c0_g1		73.19130685	6.747899416	26.68092165	-3.092140257	4.29E-09	2.32E-07
TRINITY_DN138938_c0_g1	DPYSL5	329.2418738	49.66733848	133.5396991	-2.550349915	4.63E-09	2.49E-07
TRINITY_DN131361_c0_g2	NPTX2	26.03764851	0	7.811294554	-4.861638269	4.80E-09	2.57E-07
TRINITY_DN143659_c7_g1	AKAP12	1084.329126	232.8668431	488.3055281	-2.120770175	5.07E-09	2.71E-07
TRINITY_DN146148_c1_g1		42.51191354	3.022395079	14.86925062	-3.35210482	5.43E-09	2.89E-07
TRINITY_DN140885_c0_g1	WNT10B	1466.025539	151.1312813	545.5995587	-2.979739125	5.81E-09	3.08E-07
TRINITY_DN151221_c4_g1		54.13356398	4.016687264	19.05175028	-3.297566791	6.02E-09	3.19E-07
TRINITY_DN134366_c2_g1		26.52939131	0	7.958817394	-4.838554201	6.61E-09	3.48E-07
TRINITY_DN149528_c4_g1		35.40782903	2.301248225	12.23322247	-3.426317654	7.84E-09	4.04E-07
TRINITY_DN149377_c1_g1	PLXNA4	48.70874997	7.937192695	20.16865988	-2.462161798	8.27E-09	4.24E-07
TRINITY_DN155295_c3_g1		1262.247244	200.5551426	519.0627732	-2.484888775	8.42E-09	4.31E-07
TRINITY_DN143076_c7_g1	PTDSS2	40.6536514	6.338332096	16.63292789	-2.507240532	8.97E-09	4.56E-07
TRINITY_DN145803_c9_g1		160.6180739	32.75135028	71.11136738	-2.178208124	9.97E-09	5.04E-07
TRINITY_DN154919_c0_g3	NCKAP5	89.80489538	10.11370024	34.02105878	-2.860432663	1.18E-08	5.90E-07
TRINITY_DN132712_c1_g2	ACSS3	24.97426226	0.553103396	7.879451056	-4.156591196	1.23E-08	6.12E-07
TRINITY_DN147488_c8_g1	MRC2	69.0354073	14.70685578	31.00542123	-2.135679659	1.29E-08	6.41E-07
TRINITY_DN149359_c9_g1	FREM2	28.00428298	1.305095576	9.314851796	-3.691678757	1.34E-08	6.60E-07
TRINITY_DN150980_c3_g1	KIT	389.7325651	75.92251001	170.0655265	-2.235746	1.47E-08	7.19E-07
TRINITY_DN134868_c0_g1	TMEFF1	73.57946924	10.31199515	29.29223738	-2.612484532	1.55E-08	7.54E-07

TRINITY_DN119920_c0_g1		24.11974745	1.180040174	8.061952356	-3.645272314	1.86E-08	8.90E-07
TRINITY_DN145934_c0_g2	PMEL	701.5906783	28.70814306	230.5729036	-3.817610997	1.89E-08	9.04E-07
TRINITY_DN138724_c1_g1	ASAH2B	28.34245351	1.542834243	9.582720024	-3.533973929	2.14E-08	1.01E-06
TRINITY_DN155234_c1_g1		45.6242854	3.564528631	16.18245566	-3.21252627	3.87E-08	1.73E-06
TRINITY_DN142779_c5_g1	STRBP	36.54488689	2.277765828	12.55790215	-3.410437615	4.14E-08	1.84E-06
TRINITY_DN151268_c11_g1		503.1982623	75.84284198	204.0494681	-2.528820833	4.24E-08	1.87E-06
TRINITY_DN152424_c3_g2	RELN	71.89462635	15.86461664	32.67361955	-2.076911209	4.28E-08	1.89E-06
TRINITY_DN151834_c4_g2		109.1569421	13.70301844	42.33919555	-2.728661666	4.30E-08	1.89E-06
TRINITY_DN146332_c9_g1	AGTR1	134.2491748	31.17495164	62.09721859	-2.015194527	4.39E-08	1.93E-06
TRINITY_DN138724_c0_g1	ASAH2	59.86983329	3.861972876	20.664331	-3.37795391	4.56E-08	1.99E-06
TRINITY_DN142109_c2_g1	AR	35.31794456	0.12877632	10.68552679	-4.591155439	4.59E-08	2.00E-06
TRINITY_DN152991_c5_g2	PLXNA4	63.43571694	8.737186208	25.14674543	-2.621283908	4.65E-08	2.02E-06
TRINITY_DN150080_c1_g1	AKAP12	382.4836506	68.60940298	162.7716773	-2.325160307	5.06E-08	2.19E-06
TRINITY_DN146661_c4_g1		1153.548196	143.5716835	446.5646374	-2.739531191	5.71E-08	2.44E-06
TRINITY_DN155809_c1_g1	PALM	267.1926038	51.19455573	115.9939702	-2.245575144	5.74E-08	2.45E-06
TRINITY_DN152208_c2_g4	SELP	158.728613	29.21593606	68.06973913	-2.293230157	5.89E-08	2.50E-06
TRINITY_DN145050_c0_g1	VWA3A	54.99962357	9.6852983	23.27959588	-2.335082298	6.12E-08	2.58E-06
TRINITY_DN148749_c4_g1		37.12844084	4.412122946	14.22701832	-2.794720502	6.24E-08	2.63E-06
TRINITY_DN148053_c0_g1		34.37016147	4.794941531	13.66750751	-2.599343859	6.32E-08	2.66E-06
TRINITY_DN151948_c8_g1		171.4239818	39.13033421	78.81842849	-2.024345823	6.81E-08	2.83E-06
TRINITY_DN138027_c0_g1	SLC27A6	60.13436962	11.37360611	26.00183516	-2.258223656	7.03E-08	2.92E-06
TRINITY_DN155016_c9_g3		6122.967497	1216.52637	2688.458708	-2.199125104	7.19E-08	2.97E-06
TRINITY_DN150977_c8_g1	FANCF	24.82911604	3.719630396	10.05247609	-2.527155497	7.51E-08	3.09E-06
TRINITY_DN151028_c4_g1		56.16128291	7.925600179	22.396305	-2.589218744	7.71E-08	3.16E-06
TRINITY_DN154809_c0_g5		43.16498958	1.267872922	13.83700792	-3.933526054	8.61E-08	3.49E-06
TRINITY_DN143277_c3_g1		28.26674297	4.765438732	11.81583	-2.392965369	8.93E-08	3.61E-06
TRINITY_DN146148_c1_g2		54.11564052	4.493720742	19.38029667	-3.139406666	9.12E-08	3.67E-06
TRINITY_DN144739_c1_g2	ADGRD1	64.50740539	1.041917568	20.08156392	-4.250298576	9.59E-08	3.84E-06
TRINITY_DN155809_c1_g4		255.9206229	46.4652064	109.3018313	-2.303969527	9.84E-08	3.92E-06
TRINITY_DN161583_c0_g2		20.12732915	0.155128211	6.146788494	-4.339357938	1.06E-07	4.17E-06
TRINITY_DN154292_c0_g3	FAM107A	96.48217175	2.501354661	30.69559979	-4.019588594	1.06E-07	4.19E-06
TRINITY_DN154846_c4_g1	GLI1	34.28781032	4.007490655	13.09158656	-2.771714691	1.13E-07	4.42E-06
TRINITY_DN150295_c1_g1	THAP6	22.95938397	0	6.887815191	-4.505292426	1.20E-07	4.70E-06
TRINITY_DN153039_c3_g1	DPT	7312.977668	1238.746734	3061.016014	-2.382741827	1.26E-07	4.91E-06

TRINITY_DN149461_c5_g3		865.9287335	183.3180669	388.1012669	-2.117462098	1.26E-07	4.94E-06
TRINITY_DN140443_c0_g1	TFAP2B	901.672798	163.5326782	384.9747141	-2.302156114	1.40E-07	5.41E-06
TRINITY_DN96851_c0_g2	FAM150A	62.68177605	2.283054669	20.40267108	-3.784581325	1.46E-07	5.61E-06
TRINITY_DN150292_c0_g1		37.5098878	1.207126919	12.09795518	-3.829113923	1.47E-07	5.66E-06
TRINITY_DN129344_c2_g1	GPRIN3	24.01089713	3.633386561	9.746639733	-2.513042826	1.59E-07	6.06E-06
TRINITY_DN151347_c2_g1	SLC4A1	226.0133668	44.45320907	98.9212564	-2.2013542	1.62E-07	6.16E-06
TRINITY_DN139744_c1_g1	TRABD2A	36.04249204	3.712220004	13.41130162	-2.906614098	1.79E-07	6.72E-06
TRINITY_DN155295_c1_g1		508.1109101	89.7074847	215.2285123	-2.329348928	1.95E-07	7.25E-06
TRINITY_DN143076_c8_g1	PTDSS1	79.89079162	18.23643836	36.73274434	-2.023613703	2.06E-07	7.64E-06
TRINITY_DN137441_c1_g1		31.31405633	4.941449335	12.85323143	-2.442498108	2.14E-07	7.88E-06
TRINITY_DN154022_c1_g1	WNK2	47.24525342	10.63525418	21.61825395	-2.035751569	2.17E-07	7.97E-06
TRINITY_DN151347_c3_g2	SLC4A1	156.3547954	25.89399876	65.03223776	-2.401485304	2.21E-07	8.11E-06
TRINITY_DN139259_c0_g1	COL8A1	250.2530662	40.33948312	103.313558	-2.432513481	2.27E-07	8.32E-06
TRINITY_DN153917_c10_g2		83.84569297	14.86512032	35.55929212	-2.315921816	2.29E-07	8.36E-06
TRINITY_DN154116_c7_g1	QRICH2	71.40919583	6.215145486	25.77336059	-3.060438596	2.39E-07	8.66E-06
TRINITY_DN143876_c12_g1		84.01726352	5.55718913	29.09521145	-3.314661709	2.42E-07	8.75E-06
TRINITY_DN136089_c1_g1	ZPBP	58.2271206	5.508162853	21.32385018	-2.989631235	2.46E-07	8.88E-06
TRINITY_DN144973_c5_g1	TYRP1	1287.043923	19.70561774	399.9071094	-4.231381071	2.47E-07	8.93E-06
TRINITY_DN57062_c0_g1		22.07488553	0.438781686	6.929612839	-4.016607968	2.49E-07	8.98E-06
TRINITY_DN106151_c0_g1		82.59736854	2.349440681	26.42381904	-3.907130651	2.77E-07	9.88E-06
TRINITY_DN154801_c5_g1		39.77260001	8.10340922	17.60416646	-2.149521058	3.03E-07	1.07E-05
TRINITY_DN148009_c1_g2	NEBL	368.3075847	59.74416629	152.3131918	-2.421874355	3.04E-07	1.07E-05
TRINITY_DN149144_c6_g2		90.98440336	20.33642479	41.53081836	-2.046494277	3.12E-07	1.10E-05
TRINITY_DN146303_c1_g3	CACNB3	106.494108	15.23522811	42.61289209	-2.559110347	3.20E-07	1.12E-05
TRINITY_DN74981_c0_g1	RNASE1	2080.767595	454.4318588	942.3325798	-2.071786984	3.21E-07	1.12E-05
TRINITY_DN148830_c1_g1		93.07759235	21.18714628	42.7542801	-2.01717905	3.24E-07	1.13E-05
TRINITY_DN143896_c11_g1	FAM69C	360.9155023	36.98197328	134.162032	-2.905966993	3.36E-07	1.16E-05
TRINITY_DN128386_c1_g1		41.84626193	8.858976641	18.75516223	-2.123463459	3.52E-07	1.21E-05
TRINITY_DN152091_c12_g2	MARCO	33.03908274	2.719194434	11.81516093	-3.097740427	3.86E-07	1.31E-05
TRINITY_DN137499_c1_g1		41.81312216	6.679062564	17.21928044	-2.436456028	4.17E-07	1.40E-05
TRINITY_DN147220_c10_g1		52.02579474	10.77796802	23.15231603	-2.133275093	4.43E-07	1.48E-05
TRINITY_DN148053_c1_g1		81.02153721	17.70791722	36.70200322	-2.058498445	4.59E-07	1.53E-05
TRINITY_DN144413_c1_g1	RGS22	67.78478204	2.07943468	21.79103889	-3.827765867	4.62E-07	1.53E-05
TRINITY_DN147488_c5_g1		46.35570114	3.038902692	16.03394223	-3.291107746	5.04E-07	1.66E-05

TRINITY_DN131427_c1_g1	DGKI	29.28017478	3.987725904	11.57546057	-2.59446365	5.42E-07	1.76E-05
TRINITY_DN149156_c4_g1		42.25432029	2.150318184	14.18151882	-3.461105327	5.45E-07	1.77E-05
TRINITY_DN139573_c0_g1	NR2F1	221.1197482	30.17966441	87.46168956	-2.600522544	5.59E-07	1.81E-05
TRINITY_DN147721_c0_g1	VSIG8	1033.753249	23.58362607	326.6345129	-3.985521461	5.82E-07	1.87E-05
TRINITY_DN152789_c2_g2		596.3468682	107.9255305	254.4519318	-2.287449873	5.89E-07	1.89E-05
TRINITY_DN136284_c0_g1	MICU3	15.24212762	0.269324393	4.761165361	-3.927143216	6.21E-07	1.98E-05
TRINITY_DN150292_c0_g2		78.32104808	2.821712407	25.47151311	-3.713665447	6.58E-07	2.09E-05
TRINITY_DN141780_c2_g1	LRIT1	71.59205766	3.538825012	23.95479481	-3.502796332	6.61E-07	2.09E-05
TRINITY_DN149691_c0_g1	CD55	674.7535217	101.3011935	273.3368919	-2.495771406	6.62E-07	2.09E-05
TRINITY_DN152684_c3_g1		106.320801	21.02277861	46.61218534	-2.181526998	6.62E-07	2.09E-05
TRINITY_DN155076_c1_g1	KRT17	108.5356873	4.148401693	35.46458739	-3.680056415	6.66E-07	2.10E-05
TRINITY_DN86850_c0_g1	FGFR3	20.95296914	0.88645283	6.906407722	-3.576734702	6.91E-07	2.17E-05
TRINITY_DN142896_c2_g1	S100B	90.96126482	8.886406011	33.50886365	-2.931615246	7.02E-07	2.20E-05
TRINITY_DN44699_c1_g1		139.5303371	4.295095672	44.86566811	-3.804889995	7.45E-07	2.33E-05
TRINITY_DN149377_c1_g2	PLXNA4	14.31635427	0.434372845	4.598967272	-3.716095461	7.60E-07	2.37E-05
TRINITY_DN137490_c0_g1	TSPAN10	84.11134651	10.52438001	32.60046996	-2.690049072	7.62E-07	2.37E-05
TRINITY_DN123934_c0_g1		18.70235451	0	5.610706354	-4.234585277	7.81E-07	2.42E-05
TRINITY_DN129344_c1_g1	GPRIN3	35.70435892	6.130052225	15.00234423	-2.34963287	8.04E-07	2.49E-05
TRINITY_DN149978_c7_g4	SLC39A10	34.4671387	7.724513923	15.74730135	-2.024912078	8.98E-07	2.75E-05
TRINITY_DN145414_c0_g1	CECR5	418.8197603	71.99953421	176.045602	-2.340355971	9.25E-07	2.83E-05
TRINITY_DN155837_c0_g3		193.9604052	33.97778294	81.97256961	-2.320504004	9.32E-07	2.85E-05
TRINITY_DN49890_c0_g1		66.31880599	7.232614735	24.95847211	-2.822369016	9.35E-07	2.86E-05
TRINITY_DN133594_c0_g1		39.98135919	3.245840508	14.26649611	-3.079635101	9.43E-07	2.88E-05
TRINITY_DN139476_c1_g3	LRRC3B	149.4777709	6.28727316	49.24442248	-3.597864051	9.85E-07	2.99E-05
TRINITY_DN148796_c0_g3	SLC38A4	31.52644964	7.140820107	14.45650897	-2.001399776	1.00E-06	3.03E-05
TRINITY_DN146348_c2_g1	EDAR	27.32816639	1.033155708	8.921658914	-3.625779388	1.06E-06	3.19E-05
TRINITY_DN136710_c0_g2	WIPF3	30.92905008	3.6605053	11.84106873	-2.735602494	1.07E-06	3.22E-05
TRINITY_DN145640_c2_g2	MBP	122.7207352	16.33883727	48.25340666	-2.614191372	1.08E-06	3.23E-05
TRINITY_DN152406_c6_g1		42.11718538	9.251154741	19.11096393	-2.047401513	1.09E-06	3.26E-05
TRINITY_DN137829_c0_g2		31.80346088	0.619685765	9.974818299	-3.933700464	1.12E-06	3.35E-05
TRINITY_DN155809_c1_g5	PALM	146.298241	32.73152139	66.80153728	-2.03402681	1.16E-06	3.45E-05

Supplementary Table 3. Outcomes and fibropapillomatosis tumor recurrence status of green turtles treated for eye tumors at the time of outcome recording.

Outcome	Total number	Number with regrowth at outcome
Surgery & 5-FU treated:		
Released	45	0
Still in rehabilitation	12	1
Died in care (other non-eye fibropapillomatosis complications)	20	4
Euthanized	32	15
Surgery only:		
Released (three of these turtles had eye tumor regrowth during rehabilitation period, which was removed by additional rounds of surgery prior to release)	6	0
Still in rehabilitation	3	2
Died in care (other non-eye fibropapillomatosis complications)	1	1
Euthanized	2	2

Supplementary Table 4. Transcript expression values underlying the differential expression analysis depicted in Figs 2A, 3B and 5B.

Data for Fig. 2A

Gene ID	Transcript ID	Expression values (TMM) per sample									
		mjCS1A	***mjCS1B***	***spCSR1B***	***mjVN1A***	***mjVN1C***	***mjVN2A***	***mjVN2B***	***spVN1A***	***spVN1B***	***spVN1I***
Pitx2	TRINITY_DN115109_c0_g1	0.076	0.209	0.237	7.879	6.419	6.903	5.993	7.501	6.968	6.667
Fndc1	TRINITY_DN152537_c0_g2	0.681	0.747	0.729	7.729	7.232	9.653	8.126	9.664	7.715	5.613
Cpxm1	TRINITY_DN150062_c3_g1	4.746	4.861	4.21	63.12	48.43	59.561	56.605	69.388	84.924	71.121
Unique	TRINITY_DN149008_c1_g1	0.028	0	0.028	30.807	27.281	28.473	35.565	30.18	54.365	48.324
S1pr3	TRINITY_DN152113_c0_g1	0.473	0.508	0.747	7.799	6.46	10.369	10.28	9.111	9.304	9.011
Nes	TRINITY_DN154746_c0_g1	0.321	0.279	0.218	5.405	4.115	5.662	4.223	6.742	7.458	4.194
Lrrc15	TRINITY_DN151118_c0_g1	0.605	0.249	0.303	12.048	9.7	16.232	14.236	16.996	18.555	14.484
Wnt5A	TRINITY_DN152355_c2_g1	3.527	4.452	3.425	22.968	24.081	26.592	22.085	22.38	26.494	20.065
Ncan	TRINITY_DN155020_c0_g3	0.057	0.05	0.066	3.441	2.387	2.969	2.559	2.013	2.273	2.333
Ntn3	TRINITY_DN155213_c8_g2	0.34	0.488	0.208	3.73	4.3	3.447	3.178	4.223	3.628	4.366
Sult1A4	TRINITY_DN153673_c8_g1	30.085	20.717	28.242	0.14	0.247	0.134	0.053	0.075	0.096	0.086
Krt7	TRINITY_DN144332_c0_g1	5.852	4.253	4.769	0.07	0.021	0.076	0.032	0.056	0.032	0.065
Unique	TRINITY_DN152568_c4_g1	61.257	53.606	79.778	1.147	1.018	0.382	0.832	1.648	1.067	1.043
Unique	TRINITY_DN132030_c1_g1	36.07	29.413	54.062	0.1	0.154	0.029	0.032	0.056	0.053	0.161
F10	TRINITY_DN145658_c0_g1	7.762	5.956	7.115	0.987	0.864	1.27	0.874	0.974	0.918	1.054
Unique	TRINITY_DN154564_c5_g1	6.467	5.159	9.13	0.02	0	0.019	0	0.084	0.021	0.022
Krt24	TRINITY_DN142587_c1_g1	285.751	219.056	498.547	4.318	5.071	7.199	6.718	6.377	6.573	14.441
Unique	TRINITY_DN145449_c0_g1	15.581	14.711	31.639	0	0	0	0	0.15	0.096	0.054
Lrp4	TRINITY_DN154801_c10_g1	6.722	6.155	9.272	1.586	1.728	1.614	1.109	1.508	1.462	1.355
Unique	TRINITY_DN147614_c0_g1	5787.308	4849.97	9498.633	6.672	10.678	3.38	5.407	0.843	1.088	2.14

Data for Fig. 3B

Gene ID	Transcript ID	Expression values (TMM) per sample									
		mjCS1A	***mjCS1B***	***spCSR1B***	***mjVN1A***	***mjVN1C***	***mjVN2A***	***mjVN2B***	***spVN1A***	***spVN1B***	***spVN1I***
Pitx2	TRINITY_DN115109_c0_g1	0.076	0.209	0.237	7.879	6.419	6.903	5.993	7.501	6.968	6.667
Nes	TRINITY_DN154746_c0_g1	0.321	0.279	0.218	5.405	4.115	5.662	4.223	6.742	7.458	4.194

Ncan t1	TRINITY_DN155020_c0_g3	0.057	0.05	0.066	3.441	2.387	2.969	2.559	2.013	2.273	2.333
Ntn3	TRINITY_DN155213_c8_g2	0.34	0.488	0.208	3.73	4.3	3.447	3.178	4.223	3.628	4.366
Itm2c t1	TRINITY_DN142485_c0_g1	4.028	3.058	1.741	38.726	37.588	40.446	48.457	43.496	42.958	43.528
Itm2c t2	TRINITY_DN142485_c1_g1	1.985	2.151	1.031	22.31	17.796	23.393	24.09	20.648	24.83	22.915
Myl9	TRINITY_DN151691_c0_g1	15.09	11.544	14.362	56.877	46.877	59.227	54.003	59.883	71.031	62.572
Crabp2	TRINITY_DN153313_c0_g2	18.645	19.283	12.309	169.824	157.563	187.574	153.498	193.64	209.21	133.166
Cthrc1	TRINITY_DN90102_c0_g1	0.492	0.388	0.606	11.489	10.266	14.284	16.86	22.146	28.991	29.539
Ncan t2	TRINITY_DN155020_c0_g2	0.057	0.05	0.047	2.214	1.183	2.015	1.621	1.62	1.974	1.968
Fgf13	TRINITY_DN137494_c1_g1	0.095	0.07	0.265	3.461	2.993	3.199	3.764	4.448	4.727	5.086
Fgf14	TRINITY_DN145215_c1_g1	0.142	0.199	0.284	3.78	2.633	3.17	3.85	2.257	4.012	3.247
Avil	TRINITY_DN149582_c2_g1	1.276	0.916	1.665	10.761	8.137	10.369	9.768	13.222	15.674	13.011
Lrp4	TRINITY_DN154801_c10_g1	6.722	6.155	9.272	1.586	1.728	1.614	1.109	1.508	1.462	1.355
Sfrp1	TRINITY_DN151935_c0_g1	0.463	0.608	0.407	8.627	6.604	9.672	8.254	15.357	19.26	14.678
Wnt10a	TRINITY_DN153250_c7_g1	0.756	0.807	1.088	5.256	5.298	4.87	5.108	4.635	3.895	3.452
Ntrk3	TRINITY_DN151833_c1_g1	0.151	0.139	0.038	1.745	1.79	1.642	1.429	4.064	2.091	1.237
Hey2	TRINITY_DN140624_c6_g1	2.222	2.55	2.129	14.172	13.64	15.945	15.25	27.418	27.422	23.291
Col5a2 t1	TRINITY_DN149983_c3_g1	15.014	11.783	14.239	75.268	68.366	85.838	81.58	141.313	142.287	94.1
Sox5 t1	TRINITY_DN135765_c2_g1	0.652	0.568	0.634	0.09	0.062	0.076	0.043	0.056	0.064	0.065
Gdnf	TRINITY_DN145042_c1_g1	0.038	0.02	0.019	0.768	0.772	0.964	0.8	0.571	0.63	0.473
Itga8	TRINITY_DN150006_c0_g1	0.104	0.08	0.284	1.885	1.965	2.435	1.909	3.586	3.318	3.065
Apcdd1	TRINITY_DN145309_c0_g1	6.287	9.323	8.875	37.34	35.171	40.026	32.6	54.415	57.256	52.583
Fgfr3	TRINITY_DN148274_c3_g1	34.661	31.883	36.625	3.82	4.074	2.091	1.632	4.186	4.065	4.463
Sim1	TRINITY_DN153759_c2_g1	0.388	0.568	0.407	1.825	1.81	1.795	1.408	1.873	1.75	2.495
Pcdh9	TRINITY_DN148701_c1_g3	0.047	0	0.038	1.057	1.183	1.127	1.344	1.011	1.056	0.806
Ncam1	TRINITY_DN143849_c0_g2	0.719	0.986	0.653	3.67	3.456	3.829	3.05	3.006	3.489	2.419
Bmp5	TRINITY_DN152084_c3_g1	2.411	0.916	2.337	0.09	0.072	0.057	0.128	0.047	0.075	0.108
Shh t1	TRINITY_DN149889_c8_g1	4.699	4.442	3.936	0.319	0.545	0.42	0.235	0.281	0.384	0.613
Thy1	TRINITY_DN149846_c1_g1	1.087	1.006	3.406	37.848	26.252	36.694	37.356	65.689	90.366	80.326
Plppr4 t1	TRINITY_DN153944_c0_g1	0	0	0.028	1.416	1.039	0.611	0.48	0.721	0.992	0.516
Ptk7	TRINITY_DN142006_c0_g1	5.663	4.781	4.627	22.39	19.133	25.503	22.437	36.679	41.624	33.098
Epha8	TRINITY_DN148651_c0_g1	0.038	0.11	0.104	2.334	2.088	2.101	1.418	5.188	4.215	3.506

Atoh8	TRINITY_DN145426_c1_g1	0.841	0.687	0.331	3.54	3.323	4.258	3.892	4.382	5.442	4.269
Pdgfra	TRINITY_DN154278_c1_g1	2.118	1.713	1.164	7.081	6.316	6.989	6.388	10.01	10.35	9
Cdh11	TRINITY_DN95919_c0_g1	4.349	4.661	5.507	26.379	22.045	28.492	23.919	45.622	56.467	40.862
Sdc2	TRINITY_DN57032_c0_g1	17.973	14.173	15.119	81.122	67.666	67.935	89.525	86.571	134.743	132.758
Wnt2b	TRINITY_DN144568_c7_g1	2.222	1.205	0.965	8.786	6.686	6.903	7.934	7.885	9.582	7.678
Ptch1	TRINITY_DN143947_c0_g1	6.42	5.906	4.712	1.406	1.553	0.955	0.864	1.039	1.003	0.979
Mef2d	TRINITY_DN145764_c0_g1	1.881	1.783	1.135	6.872	5.473	5.586	7.049	7.023	6.455	4.602
Prickle1 t1	TRINITY_DN154832_c2_g1	2.212	1.703	1.372	8.427	6.542	9.09	6.9	13.475	13.893	9.71
Hlx	TRINITY_DN153116_c6_g1	1.352	1.016	0.71	4.657	3.436	4.411	3.775	4.57	3.308	3.151
Nrep	TRINITY_DN104301_c0_g1	32.657	31.295	53.476	151.662	129.5	160.801	176.927	164.583	252.318	158.597
Col4a1	TRINITY_DN155321_c9_g1	4.179	2.42	2.583	15.508	13.435	15.077	14.823	29.431	27.956	18.882
Mmp14	TRINITY_DN147874_c1_g1	16.943	11.982	9.726	41.568	37.063	49.908	49.886	63.048	64.821	47.55
Ndnf	TRINITY_DN31879_c0_g1	1.494	1.116	1.306	9.534	7.18	6.77	9.427	16.631	19.142	17.366
Wnt6	TRINITY_DN151779_c0_g1	4.037	5.189	7.825	20.116	20.337	19.812	24.89	25.18	33.216	25.399
Spock1	TRINITY_DN155359_c2_g3	0.123	0.02	0.038	0.858	0.555	0.993	0.693	1.358	1.227	1.129
Col5a2 t2	TRINITY_DN149983_c3_g2	11.941	7.42	5.734	54.813	38.318	62.474	46.644	104.728	113.797	77.573
Sema5b	TRINITY_DN148106_c4_g1	0.444	0.488	0.454	1.526	1.697	1.843	1.973	3.474	3.169	2.419
Shank1	TRINITY_DN145471_c3_g1	0.227	0.139	0.038	1.715	1.913	1.786	1.61	2.182	3.393	2.086
Prickle1 t2	TRINITY_DN154832_c1_g1	2.714	1.892	2.621	8.328	8.065	8.269	7.71	15.086	12.548	9.108
Lep	TRINITY_DN134755_c0_g1	0.009	0.05	0	0.808	0.7	1.022	1.877	2.032	1.91	1.613
Itga5	TRINITY_DN152001_c7_g2	9.152	4.9	4.636	24.305	20.81	24.319	29.475	34.02	38.626	32.958
Prrx1	TRINITY_DN142151_c7_g1	4.482	2.341	1.694	15.299	12.869	14.475	14.471	23.523	25.075	21.237
Doc2a	TRINITY_DN137692_c0_g1	0	0.01	0	0.957	0.525	1.098	1.312	0.852	1.344	0.656
Satb2	TRINITY_DN133980_c0_g1	0.132	0.1	0.066	0.618	0.689	0.649	0.736	0.796	0.939	0.484
Plppr4 t2	TRINITY_DN153944_c0_g2	0.019	0.07	0.085	3.181	3.364	1.184	1.237	1.039	2.86	1.925
Dpf1	TRINITY_DN152262_c1_g1	0.227	0.239	0.293	1.486	1.327	1.442	1.653	3.362	2.433	2.624
Tgfbr2	TRINITY_DN149259_c5_g1	0.955	0.946	0.965	4.069	3.127	3.867	4.074	7.145	7.725	6.936
Tbx20 t1	TRINITY_DN155512_c0_g5	0.076	0.11	0.227	0.698	1.018	1.003	0.949	1.039	1.067	1.129
Adcyap1	TRINITY_DN142321_c1_g1	0.369	0.518	0.274	2.324	3.045	1.461	1.29	2.716	2.689	2.527

Epha5	TRINITY_DN155480_c1_g1	0.86	0.428	0.643	1.945	2.87	3.008	3.103	2.566	2.646	1.989
Fgfr3	TRINITY_DN148274_c4_g1	1.702	1.882	2.488	0.04	0.165	0.191	0.043	0.215	0.235	0.161
Tbx20 t2	TRINITY_DN155512_c0_g4	0.019	0.02	0.114	0.578	0.473	0.745	0.459	0.899	0.864	0.742
Shh t2	TRINITY_DN149889_c5_g1	1.957	1.882	2.157	0.16	0.195	0.086	0.011	0.028	0.064	0.129
Ank1	TRINITY_DN155769_c2_g2	2.572	2.859	2.091	0.977	0.442	0.659	0.693	0.403	0.331	0.366
Sema3a	TRINITY_DN148657_c0_g1	1.447	0.996	0.785	0.14	0.134	0.057	0.096	0.272	0.181	0.172
Tubb3	TRINITY_DN154106_c0_g1	39.663	43.656	34.061	9.435	7.478	13.778	9.619	6.255	5.25	6.054
Sox10 t1	TRINITY_DN151063_c0_g2	1.683	1.594	2.148	0.17	0.309	0.086	0.032	0.122	0.139	0.452
Sox5 t2	TRINITY_DN135765_c1_g1	0.917	0.548	0.36	0.04	0.021	0.01	0.011	0.047	0.032	0
Sox10 t2	TRINITY_DN151063_c0_g3	1.683	1.514	1.94	0.209	0.134	0.124	0.021	0.047	0.213	0.226
Scn3b	TRINITY_DN153068_c2_g3	0.747	0.747	0.624	0.12	0.113	0.124	0.096	0.056	0.181	0.161
Tfap2b t1	TRINITY_DN140443_c1_g1	2.25	2.829	3.926	0.389	0.627	0.124	0.245	0.459	0.534	0.462
Gli3	TRINITY_DN116096_c1_g1	0.737	0.647	0.293	0.05	0.072	0.038	0.064	0.066	0.064	0.075
Chrdl1	TRINITY_DN134272_c0_g1	22.89	7.849	1.741	0.309	0.206	0.086	0.021	0.047	0.011	0.011
Alk	TRINITY_DN150686_c7_g2	0.719	0.886	0.397	0.09	0.175	0.124	0.053	0.066	0.053	0.065
Tfap2b t2	TRINITY_DN140443_c2_g1	3.706	3.058	4.475	0.429	0.73	0.201	0.075	0.215	0.363	0.452
Slc8a1	TRINITY_DN148755_c2_g1	1.163	3.237	3.17	0	0	0	0	0.075	0.203	0
Pdgfc	TRINITY_DN137741_c0_g1	8.178	5.468	2.678	0.957	1.07	0.764	0.81	0.403	0.907	0.28
Ntm	TRINITY_DN147174_c0_g2	1.626	1.813	1.353	0.189	0.35	0.162	0.053	0.037	0.021	0.129
Dpysl5	TRINITY_DN138938_c0_g1	2.61	2.789	3.709	0.558	0.586	0.764	0.363	0.159	0.16	0.28
Wnt10b	TRINITY_DN140885_c0_g1	18.059	16.972	23.54	2.533	3.035	0.898	1.29	0.478	0.726	3.581
Kit	TRINITY_DN150980_c3_g1	1.513	1.464	3.245	0.618	0.309	0.497	0.181	0.187	0.341	0.387
Reln	TRINITY_DN152424_c3_g2	1.579	0.966	0.501	0.219	0.36	0.258	0.309	0.234	0.299	0.258
Palm t1	TRINITY_DN155809_c1_g1	2.931	1.375	0.606	0.329	0.216	0.286	0.171	0.346	0.277	0.398
Gli1	TRINITY_DN154846_c4_g1	0.917	0.787	1.694	0.1	0.082	0.162	0.053	0.3	0.096	0.065
Tfap2b t3	TRINITY_DN140443_c0_g1	5.039	4.641	8.061	1.027	1.399	0.659	0.299	0.618	1.216	1.764
Cacnb3	TRINITY_DN146303_c1_g3	1.815	1.424	0.606	0.279	0.309	0.201	0.181	0.037	0.053	0.151
Nr2f1	TRINITY_DN139573_c0_g1	3.763	1.813	1.164	0.369	0.555	0.382	0.107	0.094	0.171	0.269
Fgfr3	TRINITY_DN86850_c0_g1	0.529	0.229	0.407	0.02	0.051	0	0	0	0.021	0.022
Palm t2	TRINITY_DN155809_c1_g5	4.066	1.813	1.183	0.758	0.669	0.334	0.384	0.403	0.459	0.333

Data for Figure 5B

Gene ID	Transcript ID	Expression values (TMM) per sample									
		mjCS1A	*mjCS1B*	*spCSR1B*	*mjVN1A*	*mjVN1C*	*mjVN2A*	*mjVN2B*	*spVN1A*	*spVN1B*	*spVN1I*
Wnt2b	TRINITY_DN144568_c7_g1	2.222	1.205	0.965	8.786	6.686	6.903	7.934	7.885	9.582	7.678
Wnt5a t1	TRINITY_DN152355_c2_g1	3.527	4.452	3.425	22.968	24.081	26.592	22.085	22.38	26.494	20.065
Wnt5a t2	TRINITY_DN152355_c4_g1	4.037	5.667	3.311	26.559	23.639	29.628	20.763	26.753	29.823	25.194
Wnt6	TRINITY_DN151779_c0_g1	4.037	5.189	7.825	20.116	20.337	19.812	24.89	25.18	33.216	25.399
Wnt10a	TRINITY_DN153250_c7_g1	0.756	0.807	1.088	5.256	5.298	4.87	5.108	4.635	3.895	3.452
Wnt10b	TRINITY_DN140885_c0_g1	18.059	16.972	23.54	2.533	3.035	0.898	1.29	0.478	0.726	3.581
Ror2	TRINITY_DN145185_c1_g1	1.068	1.056	0.946	4.508	4.32	5.471	5.3	5.834	6.829	5.291
Apcdd1	TRINITY_DN145309_c0_g1	6.287	9.323	8.875	37.34	35.171	40.026	32.6	54.415	57.256	52.583
Sdc2	TRINITY_DN57032_c0_g1	17.973	14.173	15.119	81.122	67.666	67.935	89.525	86.571	134.743	132.758
Sdc3 t1	TRINITY_DN150342_c1_g1	0.492	0.319	0.378	2.812	2.51	3.246	2.367	3.221	4.012	2.86
Sdc3 t2	TRINITY_DN150342_c0_g1	1.021	0.936	0.606	3.999	3.477	4.211	3.583	5.665	7.619	4.936
Prickle1 t1	TRINITY_DN154832_c2_g1	2.212	1.703	1.372	8.427	6.542	9.09	6.9	13.475	13.893	9.71
Prickle1 t2	TRINITY_DN154832_c1_g1	2.714	1.892	2.621	8.328	8.065	8.269	7.71	15.086	12.548	9.108
Mmp7	TRINITY_DN143932_c2_g2	0.955	1.643	2.545	19.627	14.659	20.882	18.907	9.289	11.3	14.065
Sfrp1	TRINITY_DN151935_c0_g1	0.463	0.608	0.407	8.627	6.604	9.672	8.254	15.357	19.26	14.678
Sox5 t1	TRINITY_DN135765_c2_g1	0.652	0.568	0.634	0.09	0.062	0.076	0.043	0.056	0.064	0.065
Sox5 t2	TRINITY_DN135765_c1_g1	0.917	0.548	0.36	0.04	0.021	0.01	0.011	0.047	0.032	0
Sox10 t1	TRINITY_DN151063_c0_g2	1.683	1.594	2.148	0.17	0.309	0.086	0.032	0.122	0.139	0.452
Sox10 t2	TRINITY_DN151063_c0_g3	1.683	1.514	1.94	0.209	0.134	0.124	0.021	0.047	0.213	0.226
Sox14	TRINITY_DN139172_c0_g1	1.484	1.116	1.239	0.04	0.031	0	0	0	0	0
Wif1	TRINITY_DN123021_c0_g1	0.17	0.179	0.066	4.079	3.858	5.156	4.319	2.997	3.244	2.559
Ppp2r2b	TRINITY_DN144335_c0_g1	0.558	0.508	0.52	2.394	1.553	1.728	2.869	3.203	2.614	2.086

Cdh11	TRINITY_DN95919_c0_g1	4.349	4.661	5.507	26.379	22.045	28.492	23.919	45.622	56.467	40.862
Ceacam1	TRINITY_DN155724_c0_g1	1.692	1.803	1.798	8.288	9.032	8.192	6.697	9.542	16.507	11.893
Cxcl8 t1	TRINITY_DN148984_c0_g1	0.567	1.464	0.606	14.012	29.893	12.861	21.019	17.829	8.024	21.27
Cxcl8 t2	TRINITY_DN148984_c1_g1	1.664	2.331	1.022	17.732	31.22	14.427	25.284	17.576	8.387	24.194
Krt5 t1	TRINITY_DN143860_c3_g1	5.909	4.253	3.425	0	0	0	0.043	0	0.043	0
Krt5 t2	TRINITY_DN149994_c3_g2	2.619	1.882	3.595	0.539	0.525	0.497	0.267	0.459	0.437	0.28
Krt5 t3	TRINITY_DN149994_c4_g1	1.579	1.265	2.28	0.399	0.576	0.458	0.256	0.346	0.373	0.258
Krt5 t4	TRINITY_DN143860_c0_g13	433.018	331.169	157.806	0.439	1.605	0.267	0	0	0	1.441
Mmp14	TRINITY_DN147874_c1_g1	16.943	11.982	9.726	41.568	37.063	49.908	49.886	63.048	64.821	47.55
Zeb1	TRINITY_DN147245_c2_g1	0.189	0.378	0.189	5.874	5.514	4.278	7.411	7.959	5.687	4.355
Pitx2	TRINITY_DN115109_c0_g1	0.076	0.209	0.237	7.879	6.419	6.903	5.993	7.501	6.968	6.667
Cthrc1	TRINITY_DN90102_c0_g1	0.492	0.388	0.606	11.489	10.266	14.284	16.86	22.146	28.991	29.539
Lrp4	TRINITY_DN154801_c10_g1	6.722	6.155	9.272	1.586	1.728	1.614	1.109	1.508	1.462	1.355
Shh t1	TRINITY_DN149889_c8_g1	4.699	4.442	3.936	0.319	0.545	0.42	0.235	0.281	0.384	0.613
Shh t2	TRINITY_DN149889_c5_g1	1.957	1.882	2.157	0.16	0.195	0.086	0.011	0.028	0.064	0.129
Gli3	TRINITY_DN116096_c1_g1	0.737	0.647	0.293	0.05	0.072	0.038	0.064	0.066	0.064	0.075
Glis1	TRINITY_DN142164_c1_g1	0.076	0.05	0.17	2.025	1.296	2.253	2.069	4.401	4.012	2.527
Ptch1	TRINITY_DN143947_c0_g1	6.42	5.906	4.712	1.406	1.553	0.955	0.864	1.039	1.003	0.979
Ptch2 t1	TRINITY_DN150555_c2_g2	24.942	23.656	17.466	2.284	3.148	2.588	1.216	0.684	0.736	2.032
Ptch2 t2	TRINITY_DN150555_c2_g1	28.61	25.698	22.546	2.902	4.794	4.488	1.568	0.955	1.216	3.419
Gli1	TRINITY_DN154846_c4_g1	0.917	0.787	1.694	0.1	0.082	0.162	0.053	0.3	0.096	0.065
Glipr2	TRINITY_DN138110_c0_g1	5.909	4.084	5.63	23.886	20.347	27.231	28.27	32.905	50.737	43.582
Col10a1	TRINITY_DN153953_c0_g3	1.097	2.321	1.457	0.17	0.051	0.201	0.032	0.112	0	0.054
Grem2	TRINITY_DN141739_c1_g1	1.598	2.251	0.908	12.466	8.898	12.174	11.016	16.181	15.717	14.13
Nes	TRINITY_DN154746_c0_g1	0.321	0.279	0.218	5.405	4.115	5.662	4.223	6.742	7.458	4.194
Nrep	TRINITY_DN104301_c0_g1	32.657	31.295	53.476	151.662	129.5	160.801	176.927	164.583	252.318	158.597

Pdgfra	TRINITY_DN154278_c1_g1	2.118	1.713	1.164	7.081	6.316	6.989	6.388	10.01	10.35	9
Bmper	TRINITY_DN134367_c1_g1	0.076	0.02	0.019	2.453	1.718	2.368	1.962	1.03	1.046	1.086
Bmp3	TRINITY_DN152936_c5_g1	30.917	23.656	22.196	4.797	4.464	3.285	1.792	2.481	2.102	1.624
Bmp5	TRINITY_DN152084_c3_g1	2.411	0.916	2.337	0.09	0.072	0.057	0.128	0.047	0.075	0.108
Smad6	TRINITY_DN151534_c2_g2	0.804	0.558	0.596	2.284	2.603	2.559	2.431	2.669	2.379	2.559
Tgfbr2	TRINITY_DN149259_c5_g1	0.955	0.946	0.965	4.069	3.127	3.867	4.074	7.145	7.725	6.936

Supplementary Table 5. Axis labels for Figure 4 and Supplementary Figure S1.

Fig. 4A x-axis labels

Top 50 Canonical Pathways of DE transcripts, ranked by p-value (IPA)	Rank	p-value revesed from log form
Basal Cell Carcinoma (BCC) Signaling	1	1.77828E-10
Axonal Guidance Signaling	2	2.45471E-10
Human Embryonic Stem Cell Pluripotency	3	2.34423E-08
PCP pathway	4	1.09648E-07
Hepatic Fibrosis / Hepatic Stellate Cell Activation	5	7.07946E-07
Role of Osteoblasts, Osteoclasts and Chondrocytes in Rheumatoid Arthritis	6	2.5704E-06
Wnt/β-catenin Signaling	7	7.24436E-06
Sonic Hedgehog Signaling	8	9.12011E-06
Inhibition of Matrix Metalloproteases	9	4.67735E-05
Regulation of the Epithelial-Mesenchymal Transition Pathway	10	0.000114815
Bladder Cancer Signaling	11	0.000128825
Caveolar-mediated Endocytosis Signaling	12	0.000234423
STAT3 Pathway	13	0.000281838
Granulocyte Adhesion and Diapedesis	14	0.000660693
Role of Macrophages, Fibroblasts and Endothelial Cells in Rheumatoid Arthritis	15	0.000851138
Agranulocyte Adhesion and Diapedesis	16	0.001023293
Role of NANOG in Mammalian Embryonic Stem Cell Pluripotency	17	0.001230269
Glioblastoma Multiforme Signaling	18	0.001862087
Molecular Mechanisms of Cancer	19	0.002137962
Corticotropin Releasing Hormone Signaling	20	0.002691535
Paxillin Signaling	21	0.003162278
Colorectal Cancer Metastasis Signaling	22	0.003630781
Ovarian Cancer Signaling	23	0.003630781
PTEN Signaling	24	0.004897788
Sphingosine-1-phosphate Signaling	25	0.005623413
Actin Cytoskeleton Signaling	26	0.005754399
Ceramide Degradation	27	0.006025596
ILK Signaling	28	0.00676083
Clathrin-mediated Endocytosis Signaling	29	0.007585776
Superoxide Radicals Degradation	30	0.007943282

PAK Signaling	31	0.008128305
Role of Wnt/GSK-3β Signaling in the Pathogenesis of Influenza	32	0.00851138
Virus Entry via Endocytic Pathways	33	0.008912509
Sphingosine and Sphingosine-1-phosphate Metabolism	34	0.01
Leukocyte Extravasation Signaling	35	0.01
HIF1α Signaling	36	0.014454398
Phosphatidylethanolamine Biosynthesis III	37	0.017378008
Ephrin A Signaling	38	0.020892961
PPAR Signaling	39	0.020892961
Melanocyte Development and Pigmentation Signaling	40	0.025703958
Gα12/13 Signaling	41	0.02630268
TR/RXR Activation	42	0.028840315
Thyroid Cancer Signaling	43	0.030199517
tRNA Splicing	44	0.030199517
Cardiac β-adrenergic Signaling	45	0.032359366
Integrin Signaling	46	0.033113112
UDP-D-xylose and UDP-D-glucuronate Biosynthesis	47	0.034673685
Mitochondrial L-carnitine Shuttle Pathway	48	0.034673685
BMP signaling pathway	49	0.040738028
Cardiomyocyte Differentiation via BMP Receptors	50	0.042657952

Fig. 4B x-axis labels

Ingenuity Canonical Pathways with activation score

BCC Signaling
PCP pathway
Wnt/β-catenin Signaling
Sonic Hedgehog Signaling
STAT3 Pathway
Glioblastoma Multiforme Signaling
Paxillin Signaling
Colorectal Cancer Metastasis Signaling
PTEN Signaling
Sphingosine-1-phosphate Signaling

Actin Cytoskeleton Signaling
ILK Signaling
PAK Signaling
Leukocyte Extravasation Signaling
PPAR Signaling
Integrin Signaling
RhoA Signaling
Regulation of Actin-based Motility by Rho
Protein Kinase A Signaling
Signaling by Rho Family GTPases
Ceramide Signaling
RhoGDI Signaling
NF-κB Signaling
AMPK Signaling
cAMP-mediated signaling
Tec Kinase Signaling
Phospholipase C Signaling
ERK/MAPK Signaling

Fig. 4C x-axis labels

Top 25 Diseases or Functions Annotation (GO Terms)
cancer
invasion of tumor cell lines
invasion of cells
migration of prostate cancer cell lines
invasion of prostate cancer cell lines
cell movement of tumor cell lines
migration of tumor cell lines
outgrowth of cells
proliferation of neuronal cells
cell movement of skin cell lines
solid tumor
proliferation of tumor cells

proliferation of cancer cells
binding of embryonic cell lines
binding of kidney cell lines
cell movement
proliferation of connective tissue cells
migration of cells
outgrowth of neurites
growth of genital organ
migration of skin cell lines
growth of tumor
cell movement of connective tissue cells
non-melanoma solid tumor
growth of connective tissue

Fig. S1D x-axis labels

Cancer-associated Diseases or Functions Annotation	vation z-sc	p-Value
cancer	3.043	0.0000031
invasion of tumor cell lines	3.009	0.000000254
migration of prostate cancer cell lines	2.777	0.00116
invasion of prostate cancer cell lines	2.757	0.000981
cell movement of tumor cell lines	2.707	0.0000801
migration of tumor cell lines	2.524	0.0000191
solid tumor	2.18	0.00000144
proliferation of tumor cells	2.158	0.000294
proliferation of cancer cells	2.158	0.00035
growth of tumor	1.97	0.0000768
non-melanoma solid tumor	1.741	0.00000601
adhesion of tumor cell lines	1.504	0.000422
malignant solid tumor	1.491	0.00000177
cell movement of brain cancer cell lines	0.458	0.00186
neoplasia of cells	0.447	0.00000158
cell death of tumor cell lines	0.375	0.000187
apoptosis of tumor cell lines	0.327	0.0000578

vasculogenesis	0.208	0.0000138
angiogenesis	0.056	0.00000532
cell death	-0.628	0.0000153
apoptosis	-0.838	0.0000148
cell proliferation of carcinoma cell lines	-1.836	0.0000325
proliferation of kidney cancer cell lines	-2.19	0.00145
